# 3D Tumor microenvironment interaction reveals AP‐1 complex regulation and contact‐mediated reprogramming of bone marrow stromal cells in chronic lymphocytic leukemia

**DOI:** 10.1002/hem3.70199

**Published:** 2025-08-13

**Authors:** Jana Lindacher, Anne Hartebrodt, Janin Dingfelder, Pascal Lukas, Laeschkir Würthner, Simon Völkl, David B. Blumenthal, Frederik Graw, Kerstin Amann, Manuela Krumbholz, Martina Haibach, Jochen Wilke, Andreas Mackensen, Gloria Lutzny‐Geier

**Affiliations:** ^1^ Department of Internal Medicine 5, Hematology and Oncology Universitätsklinikum Erlangen, Friedrich‐Alexander‐Universität Erlangen‐Nürnberg (FAU) Erlangen Germany; ^2^ Bavarian Cancer Research Center (BZKF) Erlangen Germany; ^3^ Department of Artificial Intelligence in Biomedical Engineering (AIBE) Friedrich‐Alexander‐Universität Erlangen‐Nürnberg (FAU) Erlangen Germany; ^4^ Department of Nephropathology, Institute of Pathology Universitätsklinikum Erlangen, Friedrich‐Alexander‐Universität Erlangen‐Nürnberg (FAU) Erlangen Germany; ^5^ Pediatric Oncology and Hematology, Department of Pediatrics and Adolescent Medicine Universitätsklinikum Erlangen, Friedrich‐Alexander‐Universität Erlangen‐Nürnberg (FAU) Erlangen Germany; ^6^ Medical Care Center for Internal Medicine Oncology and Haematology Erlangen Germany; ^7^ Medical Care Center Oncology and Haematology Fürth Germany

## Abstract

Chronic lymphocytic leukemia (CLL) cells actively reprogram their tumor microenvironment (TME) to promote drug resistance and tumor progression. Tumor cell survival critically depends on heterotypic communication with benign cells in the microenvironment, particularly bone marrow‐derived stromal cells (BMSCs). Our three‐dimensional (3D) approach allows us to investigate spatially defined, mutual direct cell–cell interactions between CLL B cells, autologous T cells, and BMSCs, forming complex scaffold‐like structures reminiscent of in vivo conditions. Here, we observe that CLL B cells localized in the core regions of 3D structures upregulate the AP‐1 transcription factor complex, which confers significant protection against therapy‐induced cell death. Additionally, regulatory T cells (T_reg_) and follicular T helper cells (T_fH_) are more abundant in the core regions. CLL B cells, in turn, induce contact‐mediated reprogramming of BMSCs, resulting in a novel, distinct BMSC population with inflammation, bone immune evasion, and cancer‐associated fibroblast‐like features. Our findings reveal critical mechanisms by which CLL cells exploit the TME to drive pathogenesis and therapeutic resistance using realistic 3D setups, highlighting the potential of targeting AP‐1 and the stromal compartment in future treatment strategies.

## Graphical abstract

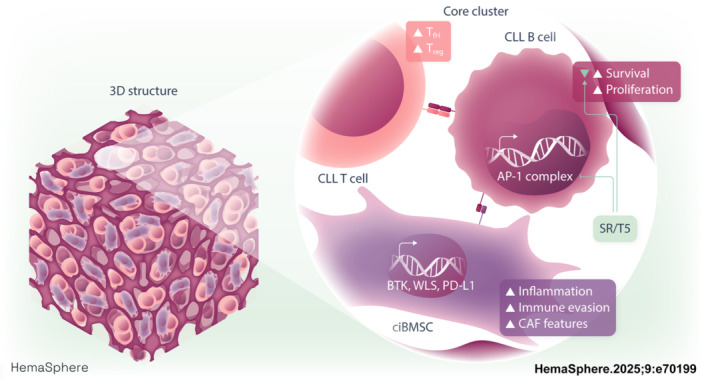

## INTRODUCTION

Chronic lymphocytic leukemia (CLL) is a complex hematological malignancy characterized by the accumulation of long‐lived malignant B lymphocytes in the peripheral blood, bone marrow, and lymphoid tissues. While significant advances have been made in understanding the molecular and cellular behavior of CLL, the complex dynamics of its tumor microenvironment (TME) remain a challenge to our understanding, particularly in the context of disease processes and therapeutic development. Within the TME, bone marrow stromal cells (BMSCs) are critical for promoting the survival of malignant CLL B cells.^1^, ^2^ BMSCs regulate multiple processes in CLL cells, including changes in metabolism,^3^, ^4^ alterations in the expression of surface,^5^ and antiapoptotic proteins,^6^ thereby contributing to drug resistance.^7^, ^8^ Notably, the bi‐directional communication between CLL cells and BMSCs leads to transcriptional and molecular reprogramming of BMSCs. Leukemic cells activate inflammatory pathways in BMSCs, such as AKT, LYN, PKCβII, and NF‐ĸB, resulting in increased tumor survival.^1^, ^9^, ^10^ The CLL‐induced changes in BMSCs resemble the molecular characteristics of cancer‐associated fibroblasts (CAFs), which are commonly observed in solid tumors.^11^, ^12^ Single‐cell analyses have identified distinct populations of inflammatory and myofibroblastic fibroblasts with potential tumor‐restraining functions in pancreatic cancer.^13^ In addition, immunomodulatory CAFs, which show enhanced MHC and interferon expression, demonstrate the capacity to transform the TME.^14^, ^15^ These findings underscore the indispensable role of BMSCs not only in shaping the pathogenesis of CLL but also in influencing disease progression and therapeutic responses by providing pro‐survival factors for CLL cells.

While two‐dimensional (2D) models have provided invaluable insights into CLL pathobiology,^1^, ^2^, ^10^, ^16^ they often fail to fully capture the cell–cell interactions that occur within three‐dimensional (3D) environments, which more closely mimic the in vivo conditions.^17^, ^18^ Especially, stromal cells, which are usually defined as spindle‐shaped cells, can grow in 3D conditions in all directions, adopting a stellate morphology.^19^ It is now evident that all cells behave differently depending on microenvironment variations by modifying their cellular shape, adhesion sites, and cytoskeletal organization.^20^ 3D cultures can preserve the spatial distribution of adhesion complexes and the interactions among different cell types, thereby more accurately reflecting cellular functions and signaling pathways. For instance, cell differentiation and the stabilization of terminally differentiated cells are better stimulated under 3D conditions.^21^ Another important aspect is that cells in 3D settings are heterogeneously exposed to oxygen and soluble factors, resembling conditions in tissues or tumoral masses.^22^ Moreover, the absence of nonmalignant microenvironmental cells can result in a loss of protection against drug treatments, leading to an altered cellular response to therapies, which is an essential consideration for successful drug screenings.^23^, ^24^ Beyond cancer research, 3D models have emerged as powerful tools in studying a wide range of diseases, including infectious diseases, as demonstrated by the use of organoids to investigate host‐pathogen interactions.^25^

In the context of hematological malignancies, 3D models have been employed to study leukemia progression and microenvironmental dependencies, including initial attempts in CLL and related disorders.^26^, ^27^, ^28^ Most studies utilize scaffold‐based matrices or hydrogel approaches to mimic a vascular niche or a bone marrow microenvironment for interactions in multiple myeloma (MM),^29^, ^30^ acute myeloid leukemia (AML),^31^ acute lymphoblastic leukemia (ALL),^32^ and diffuse large B‐cell lymphoma (DLBCL).^33^ Recently, an in vitro lymph node‐mimicking 3D model demonstrated long‐term CLL proliferation and survival using CLL:T cell ratios similar to those observed in lymph nodes.^34^ Therefore, 3D cell culture techniques have emerged as a promising tool to bridge the gap between simplified cell culture systems and animal models, offering a more physiologically relevant platform for studying disease processes.^17^ Previous work from our group has demonstrated that CLL B cells cultured in a scaffold‐based 3D model exhibit distinct spatial behavior and differential therapeutic responses, depending on their localization within the structure.^35^ In particular, cells located in the inner core show increased resistance to targeted therapies compared to those at the periphery. These findings support the notion that spatial organization within the tumor microenvironment critically influences treatment outcome and cellular phenotype. Building on this, we focused our current analysis on characterizing core‐ and periphery‐associated niches, with the aim of uncovering region‐specific features of leukemic and stromal cell interactions. This concept is supported by earlier observations using 3D co‐cultures, where distinct microenvironmental cues, such as nutrient gradients, oxygen availability, and cell density, were shown to shape leukemic cell behavior and response to inhibitors.^35^

Here, we analyze the cellular interactions between BMSCs, malignant B cells, and autologous T cells in CLL, using a fully human 3D model as an innovative platform to elucidate how these interactions contribute to therapeutic resistance and disease progression. Our 3D setting enables the visualization of cell–cell contacts, signaling cascades, and the exchange of soluble factors among BMSCs and patient‐derived immune cells within a spatially organized microenvironment. Specifically, we investigate the differences in cellular behavior and therapeutic responses between primary CLL cells localized in the inner core regions versus the periphery of the 3D structures, reflecting potential spatial heterogeneity. We have previously demonstrated that CLL cells in the inner core region exhibit altered therapeutic responses during BMSC contact compared to CLL cells cocultured in standard 2D systems.^35^ In this work, we characterize the cellular composition of these defined regions and analyze the interaction, differentiation status, and activity of patient‐derived, autologous immune cells. Our key findings reveal that CLL B cells in the core region express elevated levels of AP‐1 complex members, which confer increased drug resistance. The AP‐1 complex is known for its role in regulating cell proliferation, differentiation, and survival. In other malignancies, such as Hodgkin Lymphoma and T‐ALL, many AP‐1 components are expressed at high levels, and the associated pathways are constitutively activated.^36^, ^37^ Moreover, we identify a novel cluster of BMSCs that emerges only upon direct contact with core‐associated CLL cells. This novel stromal population exhibits a distinct transcriptomic signature, suggesting a specialized role in mediating cell–cell interactions and therapeutic resistance. By characterizing the unique features of both AP‐1‐expressing, core‐associated CLL cells and the novel, core‐associated BMSC subset, our data offer promising approaches for the development of therapeutic strategies that disrupt these critical interactions in CLL.

## MATERIALS AND METHODS

### Primary cells and cell culture

After informed patients' consent and in accordance with the Helsinki declaration, peripheral blood was obtained from patients with a diagnosis of CLL. The study was approved by the Ethics Committee of the University of Erlangen‐Nürnberg (number: 219_14B, addendum 59_17 Bc, 24‐487‐Bp_2024.12.19). PBMCs were isolated from heparinized blood samples from patients by centrifugation over a Ficoll‐Hypaque layer (Biochrom) and cryopreserved. Details on the patient samples used in each experiment are given in the patient list (Supporting Information S1: Figure 6). After thawing, cells were cultured in RPMI‐1640 medium (Gibco), supplemented with 10% fetal calf serum (ccpro), 1% l‐glutamine (Thermo Fisher), 0.6% MEM NEAA (PAN Biotech), 1% penicillin/streptomycin, 1% HEPES, 1% Na‐pyruvate, 0.2% antibiotic‐antimycotic, and 0.007% β‐mercaptoethanol (Gibco). The survival, B and T cell content, and the ratio of κ and λ light chains were analyzed. The BMSC human cell line HS‐5 (CRL‐11882; ATCC) was cultured in DMEM + GlutaMAX (Gibco) supplemented with 10% fetal calf serum and 0.4% penicillin/streptomycin and tested to be free from mycoplasma. All cell cultures were incubated at 37°C and 5% CO_2_ in a fully humidified atmosphere.

### 3D cell culture

The scaffolds (Alvetex; Reprocell Europe) are tissue‐like structures with a pore size of 36–40 µm, a surface area of 1.9 cm^2^, and a thickness of 200 µm. They were prepared according to the manufacturer's protocol and additionally coated with 0.1% gelatine for 20 min. HS‐5 BMSC cells were added to the center of the scaffold in a small volume of 70 µL in an approximate cell density of 0.4 × 10^6^ cells/cm^2^ and incubated for 1 h before medium application. After 7 days, PBMCs containing primary CLL B cells and autologous T cells were added at a total number of 2 × 10^6^ cells in a patient‐dependent ratio with B cells outnumbering T cells by at most a factor of 10 and incubated for a further 4 days. To define spatial regions, loosely attached cells were removed by gently rinsing 5–7 times in a spiral shape and collecting the entire supernatant. These cells, located primarily at the scaffold surface, were designated as “peripheral.” In contrast, cells from the scaffold interior (“core”) were obtained after cutting the scaffold into small pieces and continuous agitation in phosphate‐buffered saline (PBS) (250 rpm, 15 min, 37 °C). This two‐step isolation procedure allows spatial separation of functionally distinct cell populations, as described previously.^35^

### Flow cytometry

Cells were washed with PBS and stained with fluorochrome‐conjugated antibodies or chemical dyes (Supporting Information S1: Figure 7: antibody list) for 10 min at room temperature. For intracellular staining, cells were treated with the Fix & Perm Cell Permeabilization Kit (Life Technologies). Samples were acquired on a BD FACSCanto^TM^ ll Flow Cytometry System (BD Biosciences) and analyzed using the Kaluza Software 2.1 (Beckman Coulter).

### Immunofluorescence staining

After 4 days of co‐cultivation, the scaffold was removed, cut into pieces, and transferred to an 8‐well chamber slide (IBIDI). The cells in the scaffold were washed twice, fixed with 4% paraformaldehyde for 10 min, and permeabilized with 0.1% Triton‐X100 for 15 min at room temperature. In order to avoid unspecific binding, cells were incubated in blocking buffer for 2 h and stained with primary antibodies (Supporting Information S1: Figure 7: antibody list) under slight shaking (10 rpm) overnight at 4°C. After further washing steps, secondary staining was achieved by incubation with antibodies for 4 h under the same conditions. Nuclei were counterstained with NucGreen (Invitrogen). The 3D cultures were mounted with ProLong Glass Antifade Mountant (Invitrogen), dried overnight, and stored at 4°C. Confocal imaging was performed on a Leica Stellaris 8 (Wetzlar, Germany) using Fiji^79^, ^80^ and OMERO^81^ for visualization.

For diffusion tests, fluorescently labeled dextrans (10.000 MW; Thermo Fisher) in a concentration of 1 mg/mL were added to the 3D scaffold cultured with BMSCs for 10 days. After incubation for 30 min at 37°C, cells were fixed with 4% paraformaldehyde for 10 min, supernatant was discarded, and 3D scaffold was directly mounted with mounting medium with DAPI (Invitrogen).

### Immunohistochemistry staining

The 3D co‐cultures were fixed, dehydrated, and embedded in paraffin. Sections of a 4 µm thickness were cut on the microtome and transferred to a microscope slide. For the staining process, paraffin was removed by incubation in the drying cabinet at 60°C for 60 min. After washing steps in rotihistol, 100% ethanol, and 96% ethanol, peroxidase activity was blocked with 3% H_2_O_2_, and slides were rinsed in 70% ethanol and TRIS‐buffered saline with Tween 20 (TBS‐T). Unmasking was achieved by incubation in a steam cooker for 30 min. Proteins were blocked with universal blocking solution (Dako), followed by primary antibody staining at 4°C overnight. Slides were washed with TBS‐T and secondary antibody binding was performed for 2 h at room temperature. Finally, 3D scaffold sections were mounted with Fluoromount‐G (Life Technologies), containing DAPI for nuclei staining. A Leica Stellaris 8 and OMERO were used for fluorescent detection and visualization.

### Cytokine array

For the analysis of released cytokines and chemokines, the supernatants of the entire co‐culture of HS‐5 cells with primary T and B cells from three CLL patients and three healthy donors were collected after 4 days. Since the proportion of B cells in healthy donors is typically very low, purified B and T cells were added in equal amounts in a total number of 2 × 10^6^ cells. The PBMCs of CLL patients, containing primary CLL B cells and autologous T cells, were added at a total number of 2 × 10^6^ cells in a patient‐dependent ratio. Therefore, the B‐cell to T‐cell ratio in the malignant settings exceeds that observed under healthy conditions. Proteome Profiler Human Cytokine Array Kit (R&D Systems) was used for detection according to the manufacturer's instructions, and data analysis was performed using ImageQuant TL 10.2 software (Cytiva).

### Gene expression analysis

#### Bulk RNA sequencing

After 4 days of co‐culture, cells from five CLL patients were harvested from the peripheral and core regions of the scaffold as previously described. CLL cells were separated by anti‐CD19 DYNA beads (Invitrogen), and total RNA was isolated using the RNeasy Plus Micro Kit (QIAGEN) as recommended by the manufacturer. After quality control of the purified RNA on an Agilent bioanalyser, the mRNA was sequenced on an Illumina Novaseq. 6000 platform. For the differential abundance testing between core regions and periphery, we used the nf core/differential abundance pipeline 23.04.2.

#### Single‐cell RNA sequencing (scRNA‐seq)

BMSCs were co‐cultured with B and T cells from four CLL patients in the scaffold for 4 days. All cells were removed from the scaffold by trypsin, and immune cells were separated using anti‐CD19 and anti‐CD3 DYNA beads (Invitrogen). Dead Cell Removal Kit from Miltenyi Biotec (Bergisch Gladbach) was used to ensure that all BMSCs loaded into the scRNA‐seq platform were viable. BMSCs cultured in the scaffold alone served as a control.

In this study, there are two sequencing runs. The demultiplexed fastq files were pre‐processed using the nf‐core/scrnaseq pipeline v2.6.0 with cellranger as an aligner. The auto mode of cellranger has been used for both experiments, using V3 barcodes. We used the filtered matrix, where empty droplets had already been removed.

The prefiltered barcode matrices were analyzed using Seurat. Doublet detection was performed using scDblFinder. The datasets are pre‐processed using SCTransform (v2) on a sample level prior to integration. We used Harmony to integrate the batches and ran a standard workflow of PCA, followed by Leiden clustering, and visualized the results using UMAP. Before the differential testing, PrepSCTFindMarkers was used to account for the difference in sequencing depth between the batches. Differential gene expression testing between the clusters was done using the Wilcoxon rank sum test. Quality control plots and a comparison of the two sequencing runs are outlined in Supporting Information S1: Figure 8.

#### Interaction

To identify distinct interactions between CLL B cells located in the core and periphery and BMSC subpopulations, we used the list of differentially expressed genes in B cells between the core and periphery. Additionally, we examined the list of differentially expressed genes among the various clusters in BMSCs. We then obtained the curated protein–protein interaction (PPI) database HIPPIE (v2.3) to find putative interaction partners of the differentially expressed genes expressed in the BMSCs. To determine putative dysregulated interactions between B cells and BMSCs, we assessed each interaction in the HIPPIE database by checking whether one interaction partner was present in the list of differentially expressed genes between the core and periphery, while the other interaction partner appeared in the list of differentially expressed genes among the BMSC clusters. This analysis helps identify BMSC clusters that may preferentially interact with either core‐ or periphery‐associated B cells.

### Image analysis and quantification

Segmentation of BMSCs on the 3D scaffolds was achieved by processing the *z*‐stack of the combined phalloidin (Biolegend), and CD90 and DAPI (Invitrogen) channels using Fiji/ImageJ (v1.54j).^79^ A 3D median filter with a radius of one pixel (px) in all dimensions (*x*, *y*, *z*) was applied, followed by contrast enhancement and thresholding with a threshold *T* of *T* > 16. Subsequent image processing using morphological filters was performed to remove artifacts and enhance structural features. Finally, visualization of the 3D BMSC scaffold was done via Paraview (v5.12.0).

For the segmentation of B and T cells, three raw *z*‐stacks of different fields of view of the same scaffold were initially processed in Fiji by applying morphological filters, followed by a 3D variance filter with a radius of one pixel in all dimensions. Random slices from the *x*–*y* and *x*–*z* dimensions were manually annotated in Cellpose (v3.0.7),^82^ resulting in 481 individual cell masks based on the NucGreen channel. These cell masks were used to train a Cellpose model based on the pretrained nuclei model with a learning rate of 0.1, weight decay of 0.0001, 500 epochs, cell probability of 1.5, and estimated diameter of 6.2 px. Training was performed in Python (v3.8.18) using an NVIDIA T400 4GB GPU. Cells were then segmented via Cellpose using the trained model with an estimated diameter of 6.2 px.

The segmented cell masks were analyzed using Python (v3.11.5) with the Nyxus package (v0.7.5) to quantify CD19 and CD3 intensities for cell classification and counting. The mean pixel intensities of CD19 and CD3 for each cell mask per *z*‐stack were calculated, scaled by the sum of all intensities, and log‐transformed. The individual intensity distributions were combined to calculate the threshold values for CD19^+^ cells (40th percentile) and CD3^+^ cells (95th percentile). The threshold values were chosen asymmetrically to account for the skewness of the combined intensity distribution for CD19 and CD3, respectively. These thresholds were applied to classify and count the CD19^+^ and CD3^+^ cells in each *z*‐stack. Cell masks that were double positive for CD19^+^ and CD3^+^ were classified as co‐localized.

The amount of apoptotic B cells of four CLL patients was quantified using the Fiji cell counter. After counting, CD19^+^ cells were checked for Caspase 3 positivity, and the proportion of apoptotic B cells was determined. The amount of 3–4 *z*‐stacks per condition was averaged and compared to the control.

### Inhibitor assay

The preparation of the 3D scaffolds and seeding of BMSCs was performed as described before. Coincident to the addition of CLL B and autologous T cells, the co‐culture was treated with the inhibitors Acalabrutinib (Cayman Chemical), Enzastaurin (LC Laboratories), or the combination of SR11302 (TOCRIS) and T5224 (Hycultec). After 3 days, cells were harvested from the periphery and core region as described previously, and CLL cell survival was determined by AnnexinV (Biolegend) and Zombie Dye (Abcam) staining.

### Quantitative real‐time polymerase chain reaction (RT‐PCR)

The complementary DNA (cDNA) synthesis was performed using the SuperScript^TM^ II Reverse Transcriptase Kit (Thermo Fisher). The cDNA was prepared with the reference dye and the appropriate forward and reverse primer to be amplified in the PCR cycler. We used the QIAGEN QuantiTect Primer Assay for WLS (NM_001002292), PD‐L1 (NM_014143, XM_006716759), and glyceraldehyde 3‐phosphate dehydrogenase (GAPDH) (NM_001256799) and Brillant III Ultra‐Fasr SYBR Green QPCR Master Mix (Agilent). Quantitative real‐time PCR was carried out using a StepOnePlus^TM^ real‐time PCR system, and data were analyzed using the StepOne v2.3 software. Gene expression levels were normalized to GAPDH.

### Statistical analysis

Data processing and graphical representation were performed with GraphPad Prism version 9.5.1 (GraphPad Prism Software Inc.). Statistical analyses were determined by two‐tailed paired *t*‐test or two‐way ANOVA and accounted for multiple testing using Holm–Sidak or Tukey, according to the figure legend. Throughout the manuscript, statistical significance was defined as *P < 0.05, **P < 0.01, ***P < 0.001, ****P < 0.0001, or ns (not significant), as indicated in the figure legends.

## RESULTS

### Strong co‐localization of CLL and BMSCs in scaffold‐based 3D co‐culture promotes tumor cell survival

We developed a scaffold‐based 3D cell culture system composed of malignant B cells and autologous T cells derived from CLL patients cultured on human HS‐5 BMSCs. This system enables natural and dynamic interactions between the different cell types. Within this setup, we can distinguish between the peripheral and core regions, with CLL cells in the core exhibiting increased resistance to multiple inhibitors.^35^ Based on these observations, we propose that CLL cells gain a survival advantage by actively manipulating directly surrounding BMSCs, a process driven by enhanced, self‐organized cell–cell communication.

**Figure 1:**
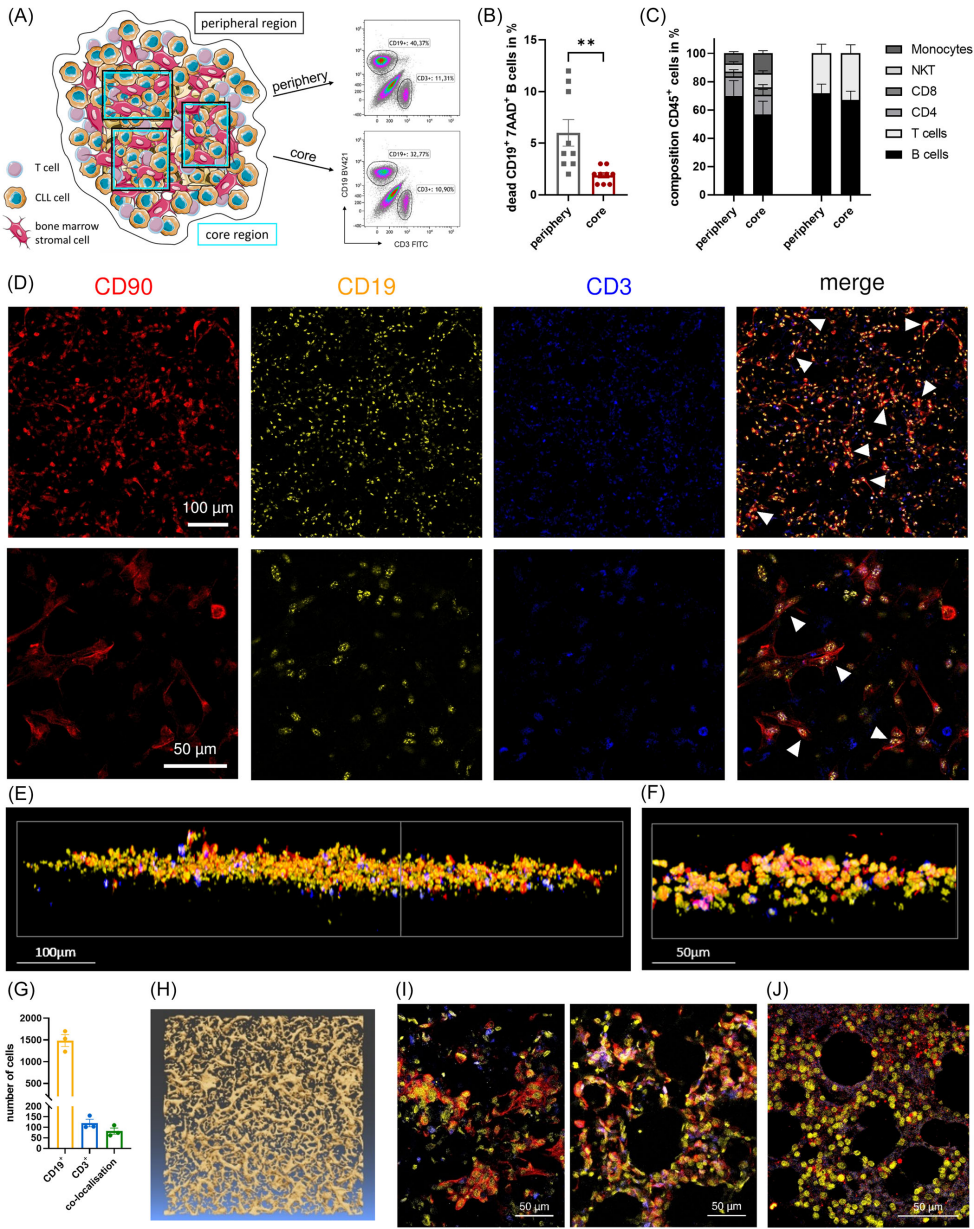
**Cellular composition and distribution of immune cells and BMSCs in the scaffold‐based 3D co‐culture**. **(A)** Illustration of the 3D model composed of BMSCs and primary autologous T and B cells derived from CLL patients. The amount of T and CLL B cells after 4 days of co‐culture in 3D cell culture conditions was measured via FACS analysis in the periphery and the core region. In the representative plot, CD19^+^ B cells and CD3^+^ T cells, as well as BMSCs, are shown. **(B)** CLL cell survival of different patients was analyzed after 4 days of co‐culture using 7AAD staining in the periphery and core region (*n* = 9), **P < 0.01 (paired *t*‐test). **(C)** Cellular composition of CD45^+^ PBMCs (left panel, *n* = 7) and the amount of B and T cells (right panel, *n* = 12) in the periphery and core region after 4 days of 3D co‐culture. **(D)** Confocal microscopy images of the 3D model. BMSCs (CD90, red), B cells (CD19, yellow), and T cells (CD3, blue) cultured in the scaffold for 4 days show a strong co‐localization of CLL and BMSCs (merge, white arrows). **(E, F)** 3D reconstruction of an immunofluorescence staining of the scaffold visualizes the distribution of the different cell types in the 3D model. *Z*‐stacks of the stained 3D cultures, including BMSCs (CD90, red), B cells (CD19, yellow), and T cells (CD3, blue), were combined using Fiji Software (number of stacks: G 145, H 211; size of stacks: G 1.06 µm, H 0.34 µm). **(G)** Quantitative image analysis of three representative *z*‐stacks of the 3D scaffold reveals the distribution of the different cell types. A cellpose‐based 3D segmentation pipeline trained on nuclear morphology was used to classify cells based on log‐transformed CD19 and CD3 intensity thresholds. Cells that were double‐positive or directly adjacent were defined as co‐localized, indicating a high proportion of T cells in close contact with CLL B cells. **(H)** 3D illustration of the spatial distribution of BMSCs. Phalloidin staining of BMSCs, grown in the scaffold for 10 days, displays a scaffolding formation of the BMSCs. **(I)** Immunohistochemical staining of a longitudinal (right panel) and a cross section (left panel) of a paraffin‐embedded 3D co‐culture suggests a strong co‐localization of CLL cells and BMSCs. B cells (CD19) are shown in yellow, T cells (CD3) in blue, and BMSCs (CD90) in red. **(J)** Immunohistochemical staining of a bone marrow biopsy from a CLL patient with B cell infiltration reveals a high similarity in structure and distribution of immune cells to the 3D model. B cells (CD19) are marked in yellow, T cells (CD3) in blue, and BMSCs (CD90) in red. Bars indicate the standard error of the mean. BMSC, bone marrow stromal cell; CLL, chronic lymphocytic leukemia; NKT, natural killer T cells.

Using FACS analysis, we demonstrate that both malignant B cells and T cells from CLL patients infiltrate the peripheral and core regions (Figure 1A). Given that approximately 90% of the CD19^+^ B cells express CD5, additional co‐staining for this CLL‐associated marker was omitted in subsequent experiments (Supporting Information S1: Figure 1A). As previously observed in 2D BMSC co‐cultures, CLL B cells exhibit a high survival rate due to contact with BMSCs.^1^, ^35^ This is also the case under 3D conditions.^35^ However, in the core regions of the 3D structure, CLL B cells show an even greater survival advantage, with reduced cell death compared to those in the periphery (Figure 1B). This regional distinction within the scaffold reflects not only physical positioning but also functional differences. As previously described, CLL cells localized in the inner scaffold regions exhibit reduced drug sensitivity and distinct gene expression profiles compared to those in the periphery.^35^ In line with this, we now observe significantly increased cell survival in the core (Figure 1B). The separation into core and peripheral regions is further supported by the extraction dynamics. Loosely adherent cells from the outer scaffold layers are removed by gentle washing, while core‐resident cells require mechanical disruption for recovery, suggesting stronger integration into the microenvironment. This spatially defined survival pattern underscores the biological relevance of region‐specific analyses within the 3D model. As expected, the analyses of the cellular composition of CD45^+^ peripheral blood mononuclear cells (PBMCs) identify B cells as the predominant cell type in both regions. In addition, CD4^+^ T cells and monocytes constitute the second‐largest populations, whereas CD8^+^ T cells and natural killer (NK) T cells account for only a small proportion (Figure 1C, left panel). The B cell‐to‐T cell ratio reveals a higher proportion of B cells in both regions (Figure 1C, right panel), which is in line with the initial B cell‐to‐T cell ratio observed in patient samples before their addition to the 3D cell culture system (Supporting Information S1: Figure 1B).

Representative immunofluorescence images of the 3D co‐culture indicate strong co‐localization of BMSCs and CLL cells, with multiple CLL cells often attached to an elongated BMSC (Figure 1D, white arrows in merge plots). Consistent with the observed B cell‐to‐T cell ratio (Figure 1C), the number of T cells is remarkably lower (Figure 1D). To visualize the 3D arrangement of BMSCs, as well as B and T cells, *z*‐stack data were reconstructed into a 3D representation (Figure 1E,F). Again, the co‐localization of BMSCs and CLL cells, as well as the potential of all three cell types to infiltrate the 3D scaffold in all dimensions, is evident. Analysis of the viability of CLL cells and BMSCs in the 3D co‐culture revealed a low number of apoptotic cells, predominantly among the malignant B cells (Supporting Information S1: Figure 1C). Moreover, scattered CLL cells, especially in cellular clusters, exhibited signs of hypoxia, which is in line with the assumption of limited oxygen diffusion into the scaffold (Supporting Information S1: Figure 1D). Quantitative image analysis confirms the predominance of the CLL B cells and demonstrates that most T cells are in close contact with B cells (Figure 1G). To quantify this spatial proximity, we employed a Cellpose‐based 3D segmentation pipeline trained on nuclear morphology and classified cells based on log‐transformed CD19 and CD3 intensity thresholds. This allowed automated identification and spatial mapping of B and T cells within the scaffold. Cells that were double‐positive or directly adjacent were classified as co‐localized, revealing a high proportion of T cells in close contact with CLL B cells across different scaffold depths (Figure 1G). Interestingly, reconstructing the BMSC distribution based on phalloidin expression reveals a distinct scaffold‐like structure in all dimensions, indicating that BMSCs firmly adhere to the scaffold and align along its structure (Figure 1H). The comparison of BMSCs cultured in the 3D scaffold for 20 h and 10 days emphasizes both their expansion and a morphological transition toward a stellate shape (Supporting Information S1: Figure 1E).

Confocal microscopy images of paraffin‐embedded sections of the 3D co‐culture confirm the cell distribution and additionally visualize the specific porous structure of the 3D scaffold (Figure 1I). A comparison of immunohistochemical staining from sections of the 3D model with a paraffin‐embedded, B‐cell‐infiltrated bone marrow biopsy from a CLL patient highlights a striking similarity, particularly in the structural organization and the close proximity of CLL cells and BMSCs (Figure 1J).

To investigate the secretion profile of the entire 3D co‐culture, supernatants were analyzed for cytokines and chemokines and compared to equally treated co‐cultures derived from healthy donors (HD) as controls. We observe increased levels of CCL2 (CC‐chemokine ligand 2), MIP1α (small inducible cytokine A3), CCL5, GM‐CSF (granulocyte macrophage colony‐stimulated factor 2), and IL1α in the malignant setting (Supporting Information S1: Figure 1F). Since no significant differences were detected, the enhanced survival of CLL cells does not appear to be primarily driven by the soluble milieu but rather by direct cell‐cell communication, which is consistent with previous publications.^1^, ^2^, ^38^

### Enhanced CLL–BMSC interactions coincide with increased T‐cell activation and senescence in the core region

**Figure 2:**
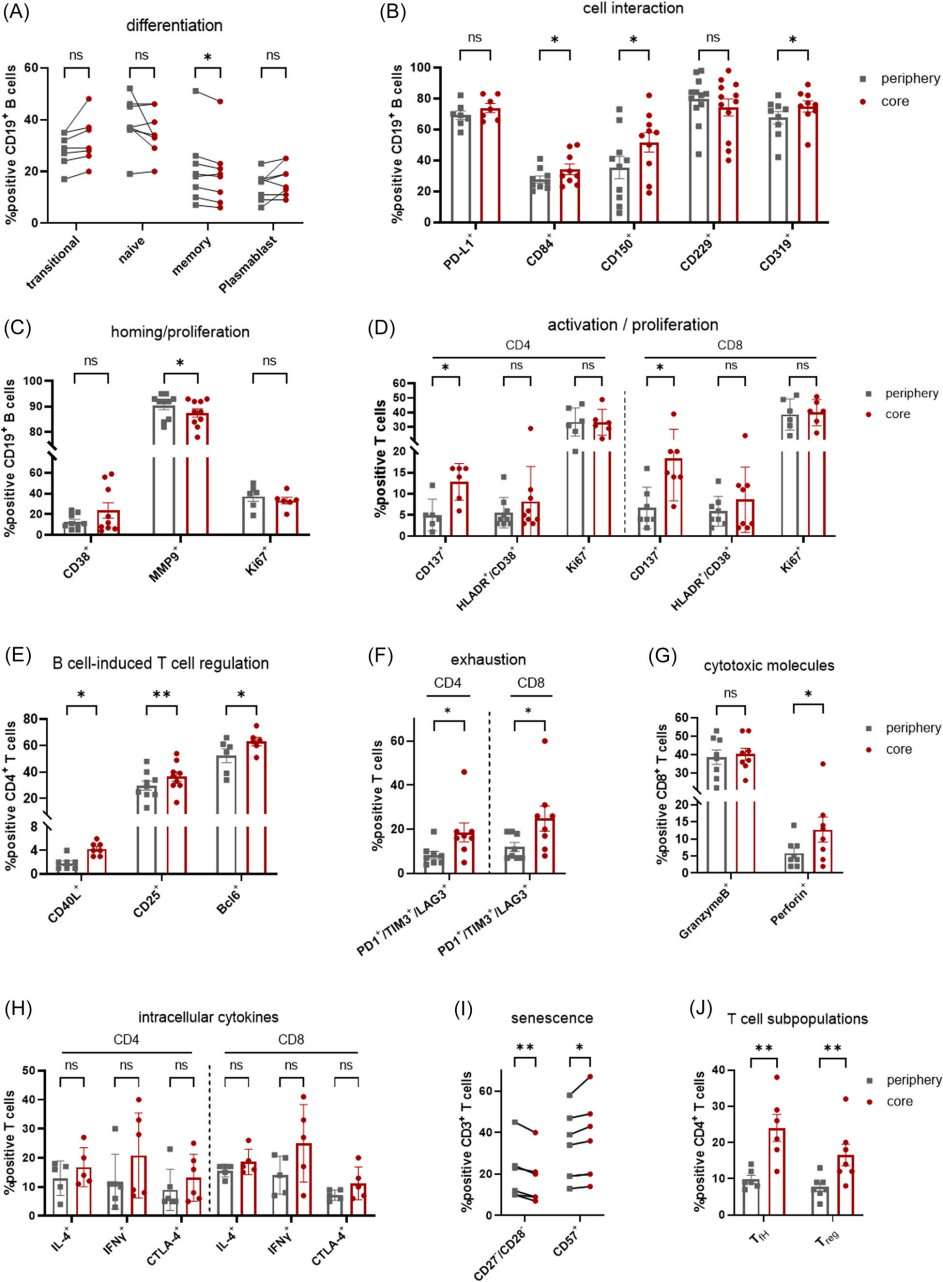
**Characterization of malignant B cells and T cells from CLL patients cultured in the 3D system**. **(A)** Visualization of the distribution of differentiation stages of B cells in the periphery and the core region after 4 days of co‐culture in the scaffold during BMSC contact (*n* = 8). Transitional (CD27^−^/CD38^+^), naïve (CD27^−^/CD38^‐^), memory (CD27^+^/CD38^−^), and plasmablast (CD27^+^/CD38^+^) CD19^+^ B cells were determined using FACS analysis. **(B)** The interaction with other cell types (*n* = 7–12) and **(C)** the homing of CLL cells (*n* = 7–11) were analyzed in the periphery and the core region after 4 days of 3D co‐culture. After 4 days of 3D co‐culture **(D)** the activation and proliferation of CD4^+^ and CD8^+^ T cells (*n* = 6–9), **(E)** the B cell‐induced regulation of CD4^+^ T cells (*n* = 5–9), (**F**) the exhaustion (*n* = 8), **(G)** the expression of cytotoxic molecules in CD8^+^ T cells (*n* = 8), **(H)** the cytokine secretion of CD4^+^ and CD8^+^ T cells (*n* = 5‐6), **(I)** the expression of different senescence marker in CD3^+^ T cells (*n* = 7–8), and **(J)** the percentage of follicular T helper cells (T_fH_; *n* = 6) and regulatory T cells (T_reg_; *n* = 7) were compared in the peripheral and the core regions. Bars indicate the standard error of the mean. *P < 0.05, **P < 0.01 (paired *t*‐test, Holm–Sidak). CLL, chronic lymphocytic leukemia.

To gain insight into the characteristic alterations of CLL B cells and autologous T cells induced by cultivation in different regions of the 3D model, the expression of several markers was analyzed using FACS. The distribution of differentiation stages of CD19⁺ cells in the peripheral and core regions of the 3D co‐culture system was examined. While memory B cells and plasmablasts account for only a small proportion, we observed a higher abundance of transitional B cells, and a phenotype resembling naïve B cells represented the largest fraction. Since all four differentiation stages are present, mature B cells are also able to grow in the 3D co‐culture. Comparing the two regions of the 3D model, only memory B cells show a significant difference, with an increased proportion in the periphery (Figure 2A). The differentiation stages of CD19^+^ cells in the PBMCs before addition to the 3D culture system are shown in Supporting Information S1: Figure 2A.

While the interaction markers PD‐L1 (Programmed cell death ligand‐1) and CD229 showed no differential expression between peripheral and core regions, the increase in CD84, CD150, and CD319 positive cells implicates a stronger communication of CLL cells with BMSCs and T cells in the core region (Figure 2B). The proportion of cells expressing the homing marker MMP9 (matrix‐metalloproteinase‐9) was higher in the periphery compared to the core region. The expression of Ki67^+^ cells indicates that CLL cells are able to proliferate in both regions, which aligns with the high survival rate of CLL cells (Figure 1B). Additionally, the proliferation rate of CLL B cells is confirmed by the co‐localization of CD19 and Ki67 observed in an immunofluorescence staining of the 3D co‐culture (Supporting Information S1: Figure 2B). CD38, an important marker of B cell survival associated with adverse clinical outcomes,^39^ was equally expressed on CLL B cells in both regions (Figure 2C).

Analysis of CD4 and CD8 T cell activation revealed an equal frequency of HLA‐DR^+^/CD38^+^ cells in CD4^+^ and CD8^+^ T cells in the periphery and the core regions. In contrast, the expression of CD137 was significantly increased in CD4^+^ and CD8^+^ T cells in the core. In addition, both T cell populations proliferated equally in the peripheral and the core regions (Figure 2D). Furthermore, elevated levels of CD40L, CD25, and Bcl6 (B‐cell lymphoma 6) were observed in CD4^+^ T cells in the core region (Figure 2E). Exhausted T cells, defined by PD1, TIM3, and LAG3, showed a trend of activation in CD4^+^ and CD8^+^ T cells in the core region (Figure 2F). The functional activity of cytotoxic T cells was demonstrated by the expression of Granzyme B and an increase of Perforin in the core region (Figure 2G). Moreover, the CD4^+^ and CD8^+^ T cell functionality is supported by cytokine secretion and the expression of the co‐inhibitory receptor CTLA‐4 with a slight trend toward higher levels in the core region, especially for IFNγ (Figure 2H). The number of CD57‐expressing T cells lacking CD27/CD28 expression was higher in the core region compared to the periphery, indicating a highly differentiated effector memory T cell phenotype with retained proliferative capacity^40^ (Figure 2I,D). Analysis of different T cell subpopulations revealed an increased abundance of follicular T helper cells (CD4^+^/CD45RO^+^/CXCR5^+^/PD1^+^) and regulatory T cells (CD4^+^/CD25^+^/FoxP3^+^) in the core region (Figure 2J). Overall, T cells in the core region appeared more activated and exhibited signs of exhaustion and senescence. However, core‐located B cells were characterized by enhanced cell‐cell interaction, and mature B cells could be maintained in the 3D model.

### RNA sequencing reveals upregulation of AP‐1 complex components in core‐associated CLL cells compared to the periphery

**Figure 3:**
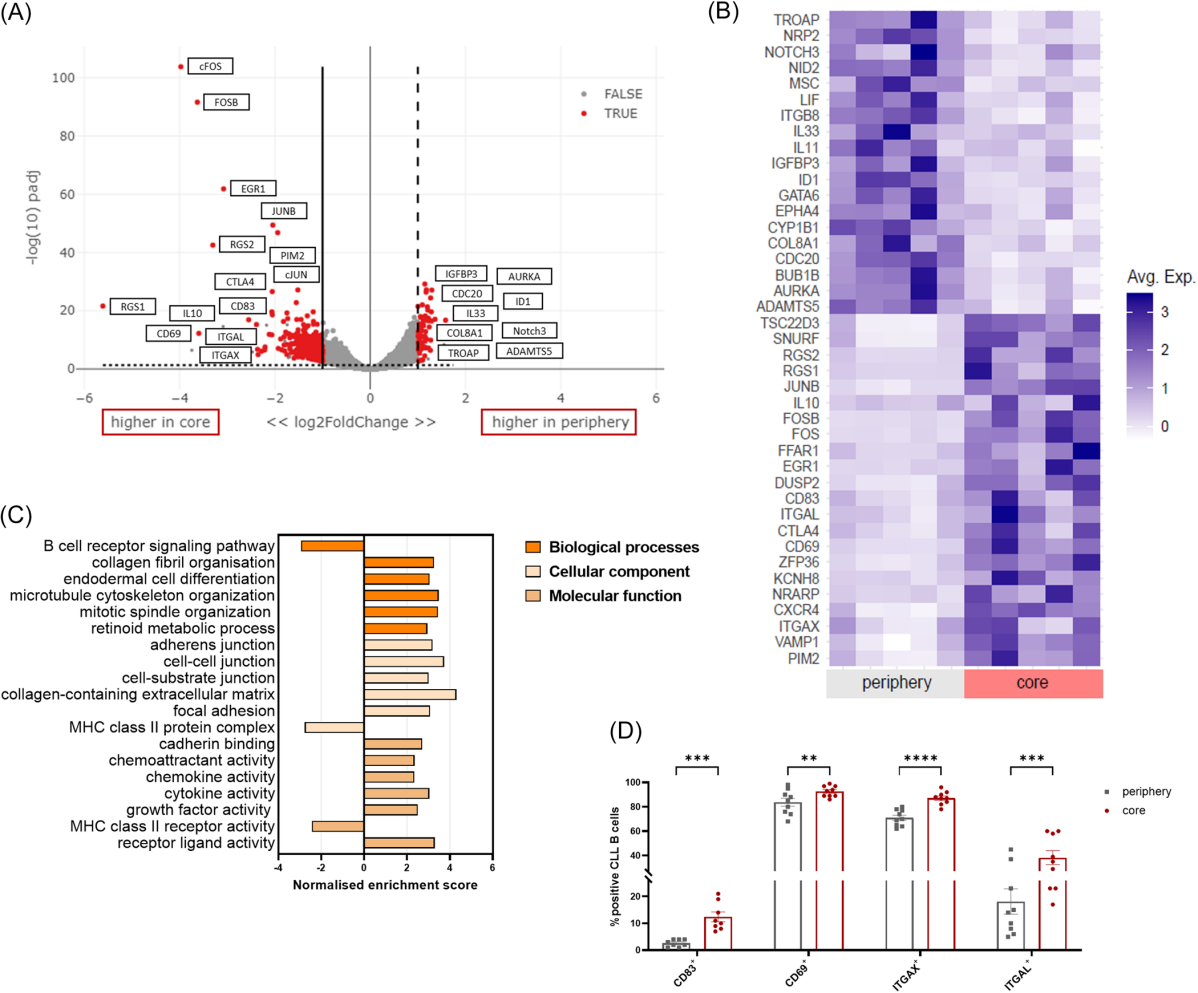
**Comparison of gene expression in CLL cells in the periphery and core region of the 3D culture system by bulk RNA sequencing**. After 4 days of 3D co‐culture, the B cells from CLL patients from the periphery and the core region were isolated, and the RNA was sequenced (*n* = 5). **(A)** Volcano plot showing relative gene expression in CLL B cells. Statistically significant upregulated genes in the core (left site) or the periphery (right site) with an adjusted P value smaller than 0.05 are marked in red. **(B)** Heatmap analysis of upregulated and downregulated genes in the periphery and the core region. Individual values of five patients are shown. Light purple indicates a lower and dark purple a higher average gene expression. **(C)** Pathway analysis of CLL B cells in the periphery compared to the core region as assessed by gene set enrichment analysis. Positive values of the normalized enrichment score (NES) show an upregulation in the core region. **(D)** Expression of several markers found to be differentially expressed in the RNA sequencing was detected in the peripheral and the core regions after 4 days of co‐culture by flow cytometry (*n* = 8–9). Bars indicate the standard error of the mean. **P < 0.01, ***P < 0.001, ****P < 0.0001 (paired *t*‐test, Holm–Sidak). Avg. Exp., average expression; CLL, chronic lymphocytic leukemia; NES, normalized enrichment score.

Bulk RNA sequencing of CLL B cells located in the periphery or the core region of the 3D co‐culture was performed to investigate differences in the gene expression of malignant B cells between both regions. This analysis revealed 74 genes upregulated in the periphery and a distinct higher number of 715 genes upregulated in the core region compared to the respective other region (Figure 3A). The highly expressed genes in the periphery include IGFBP3, Notch3, and ID1, which play key roles in proliferation and apoptosis. In addition, genes involved in cell cycle regulation, such as AURKA (aurora kinase A) or CDC20 (cell division cycle 20), as well as genes crucial for the migration and cell adhesion, including COL8A1 (collagen type VIII alpha 1 chain), ADAMTS5 (ADAM metallopeptidase with thrombospondin type 1 motif 5), and TROAP (trophinin‐associated protein), were upregulated in the periphery. Moreover, the expression of IL33 suggests an activation of the inflammatory response in the periphery.

In the core region, we observed an upregulation of the immune system regulators IL10 and CTLA4, as well as signal transduction inhibitors RGS1 and RGS2 (regulator of G‐Protein signaling 1/2). Furthermore, genes associated with proliferation and survival, including CD69, EGR1 (early growth response protein 1), and PIM2 (proviral integrations of moloney virus 2), were increased, suggesting a potential role in tumor progression. Enhanced cell‐cell interaction and adhesion were indicated by the upregulation of CD83, ITGAX, and ITGAL (integrin subunit alpha X/L). Notably, several key components of the AP‐1 complex were highly expressed in the core region, including cFos (FBJ murine osteosarcoma viral oncogene), FosB, cJun (V‐Jun avian sarcoma virus 17 oncogene homolog), and JunB. Activation of the AP‐1 complex supports the proliferation and survival of CLL cells. The gene expression levels of selected genes of the individual samples are visualized in the heatmap (Figure 3B).

Gene set enrichment analysis (GSEA) identified several biological processes, cellular components, and molecular functions that are differentially regulated in the core region compared to the periphery. These include cytoskeletal organization and extracellular matrix remodeling, as well as increased cell–cell and cell–matrix interactions mediated by various cell junctions and enhanced communication through multiple signaling pathways. In contrast, B cell receptor signaling and MHC class II pathways were upregulated in the periphery (Figure 3C). The differential expression of several genes was validated at the protein level by FACS analysis (Figure 3D). Thus, core‐localized CLL cells can be characterized as CD83^+^/ITGAX^high^ cells that overexpress key components of the AP‐1 complex.

### Single‐cell RNA sequencing reveals a novel BMSC subpopulation

**Figure 4:**
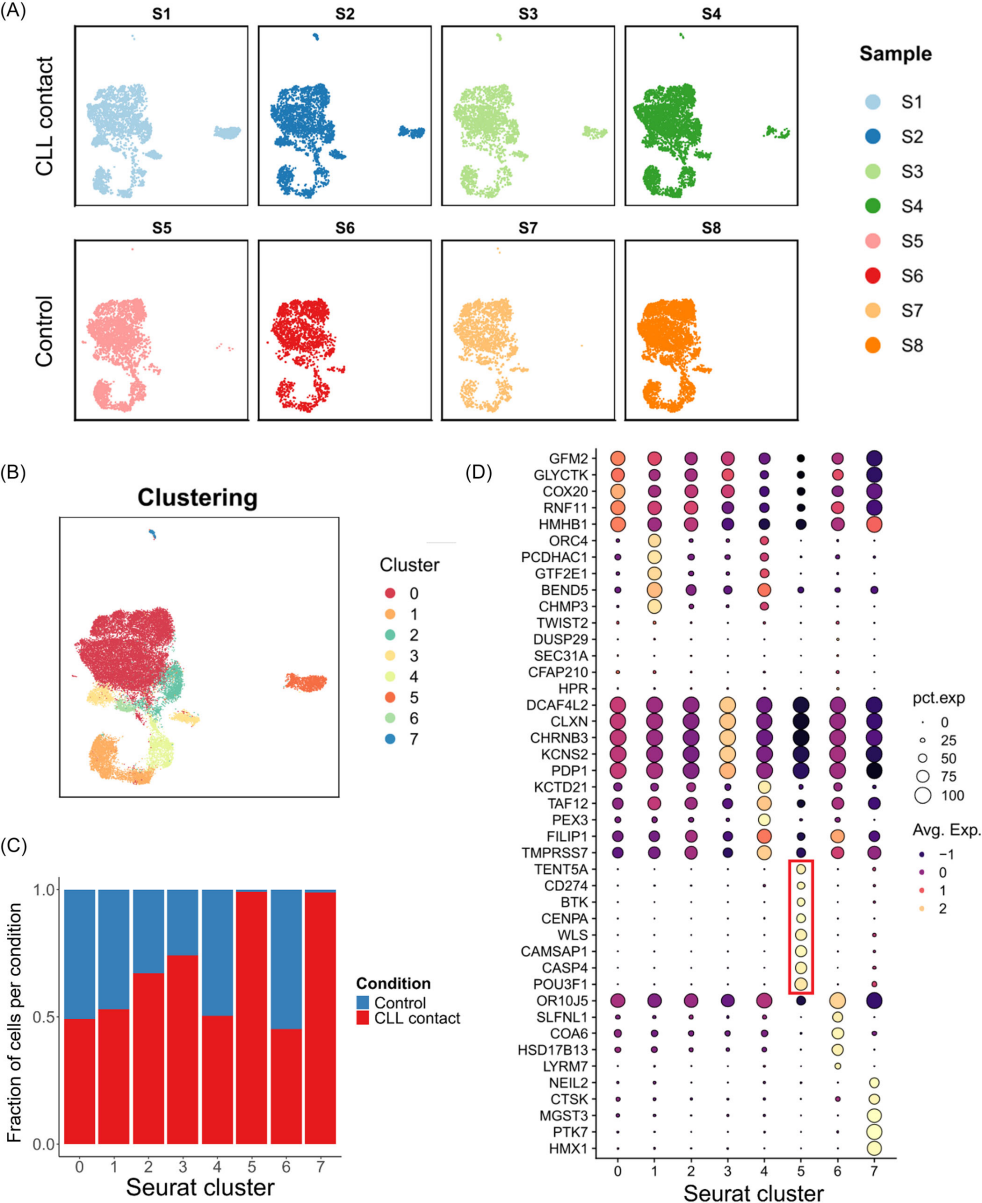
**Single‐cell RNA sequencing of BMSCs was performed after contact with CLL cells for 4 days in the 3D cell culture system**. BMSCs cultured in the 3D scaffold without any other cell types served as a control. **(A)** Integrated UMAP projections of all eight samples are visualized in separate plots. While the samples S1‐S4 had been in contact with CLL B cells, the samples S5–S8 represent BMSCs cultured alone in the 3D scaffold. **(B)** The clustering of all samples revealed eight clusters (cluster 0–7). Cell count in different clusters: Cluster 0 (15,094 cells), cluster 1 (3430 cells), cluster 2 (2032 cells), cluster 3 (1605 cells), cluster 4 (1543 cells), cluster 5 (1350 cells), cluster 6 (472 cells), and cluster 7 (161 cells). **(C)** Comparison of cells with CLL contact and the control cells uncovers cluster 5 as a novel cluster, only appearing after BMSCs had been in contact with CLL cells. **(D)** Heatmap displays genes that are differentially expressed in each cluster compared to all other clusters. While the size of the circles indicates the number of cells expressing the genes in percent, the intensity of the average expression is encoded by the color. Avg.Exp., average expression; CLL, chronic lymphocytic leukemia cells; pct.exp, percent expression.

The gene expression of the BMSCs was analyzed by single‐cell RNA sequencing. BMSCs, and T and B cells from four CLL patients were co‐cultured in the 3D scaffold for 4 days, as described before. Afterward, T and B cells were depleted using CD3 and CD19 beads, and the RNA was isolated from the remaining BMSCs for further analysis. To determine the gene expression changes induced by CLL contact, these samples were compared to BMSCs cultured alone in the 3D system, without interaction with other cell types. Integrated UMAP projections of each sample revealed a high similarity of the BMSCs cultured in the scaffold either with CLL contact (S1, S2, S3, S4) or without (S5, S6, S7, S8) contact to other cell types (Figure 4A).

Based on gene expression profiles, embedded clustering of all eight samples revealed eight distinct cell clusters (cluster 0–7) (Figure 4B). Comparing the two culture conditions, it is striking that the clusters 7 and 5 emerged only in the presence of CLL cells in the co‐culture (Figure 4C). Cluster 7 (161 reads) results due to the experimental design, as we add PBMCs to the 3D culture system, but are only excluded for T and B cells. Therefore, cluster 7 represents the remaining monocytes. Cluster 5 (1350 reads) exclusively consists of BMSCs that had direct contact with CLL B cells and was therefore designated as ciBMSCs (contact‐induced BMSCs). To rule out the possibility that cluster 5 might have arisen due to statistical or technical artefacts, we computed various quality metrics for all clusters, outlined in Supporting Information S1: Figure 3. Within the data, the sequencing depth was comparable to other clusters, and we did not observe significant differences in cell cycle phase distribution or doublet score.

For further characterization, the gene expression was compared between all eight clusters, and a selection of differentially expressed genes is presented in Figure 4D. In cluster 5, we observed increased expression of POU3F1 (POU class 3 homeobox 1) and TENT5A (terminal nucleotidyltransferase 5A), both involved in transcriptional and translational processes. Additionally, microtubule organization and chromatin structure were among the most highly regulated biological processes in cluster 5, driven by the upregulation of CAMSAP1 (calmodulin‐regulated spectrin‐associated protein 1) and CENPA (centromere protein A). The upregulation of SMO (smoothened) and LYN suggests that ciBMSCs exhibit features of chondrocytes and CAFs, potentially contributing to tumor progression by remodeling the extracellular matrix or secreting cytokines.^41^, ^42^ Moreover, high levels of BTK (Bruton tyrosine kinase) in ciBMSCs could promote cancer cell growth and progression.^43^ Of note, the regulation of CASP4 (Caspase 4), along with the upregulation of PD‐L1 (CD274) and WLS (WNT ligand secretion mediator) in cluster 5, suggests a mechanism by which ciBMSCs may locally suppress immune responses.^44^, ^45^ Furthermore, the plasticity of reprogrammed ciBMSCs was assessed to determine whether this stromal subset represents a transient or stable state. For this purpose, immune cells were removed by intensive flushing after 4 days of co‐culture, and protein expression of selected markers previously identified as upregulated in cluster 5 was assessed by flow cytometry after an additional 3 days. While the proportion of PD‐L1^+^ and POU3F1^+^ BMSCs remained stable following CLL cell removal, a clear reduction in the expression of BTK and WLS was observed after 7 days. These findings suggest that CLL contact induces both reversible and persistent changes in the characteristics of ciBMSCs (Supporting Information S1: Figure 4).

### PPI analysis of RNA sequencing data reveals putative interactions between core‐located CLL cells and ciBMSCs

**Figure 5:**
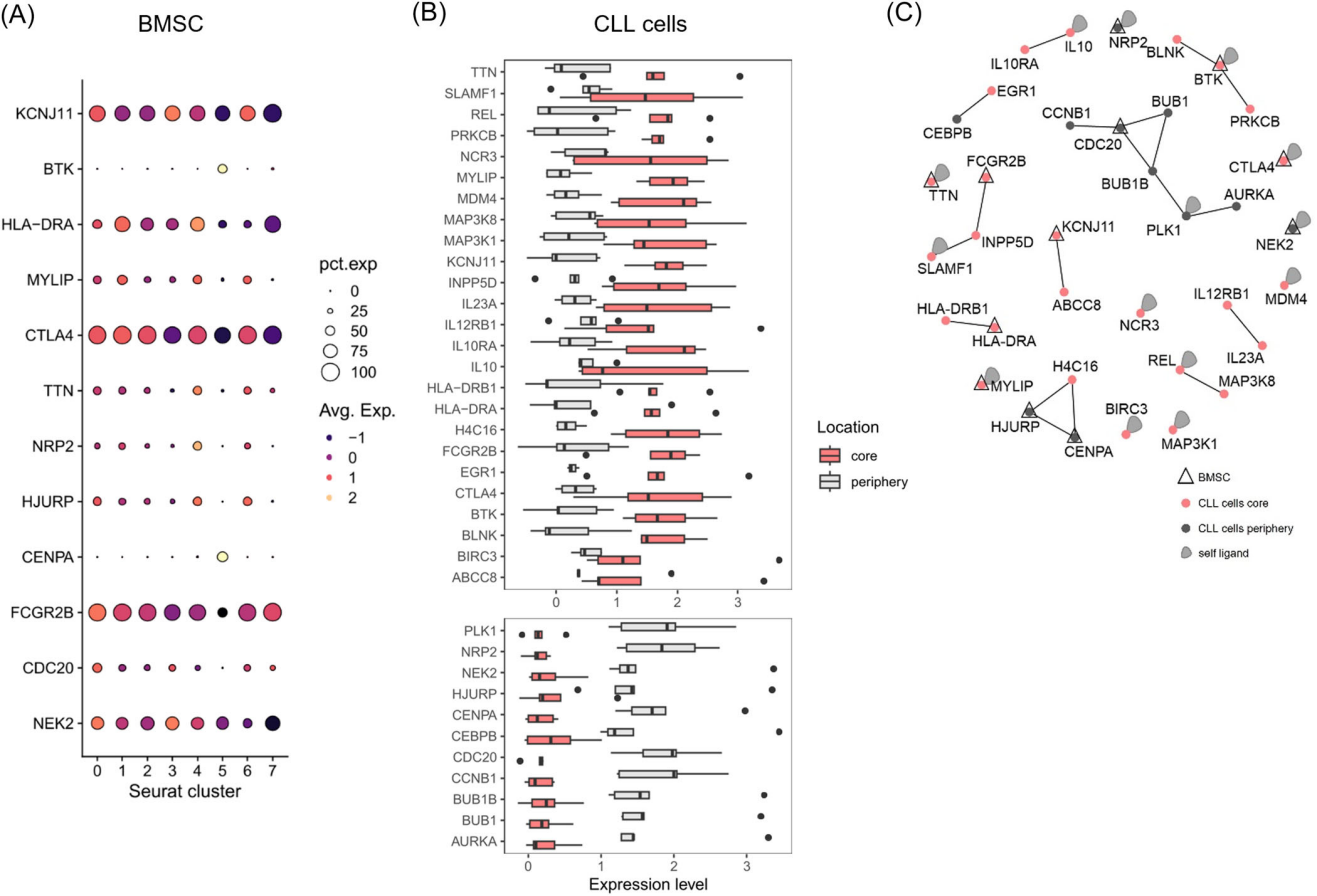
**Protein–protein interactions between BMSCs and CLL B cells in which one interaction partner was differentially expressed were extracted from HIPPIE**. **(A)** Expression level of putative interaction partners in BMSCs that are differentially expressed between any of the clusters and present in at least 10% of the cells in any cluster. The number of cells expressing the genes in percent is indicated by the size of the circles, and the intensity of the average expression is encoded by the color. **(B)** Differentially expressed genes in CLL B cells of the core (red) or in the periphery (gray). Upregulated genes in the core region are shown in the upper panel, and upregulated genes in the periphery in the lower panel. **(C)** Network of selected putative interactions of proteins with other proteins (solid lines) or with themselves (squiggly lines). Proteins present in BMSCs are depicted as a triangle and as a circle in CLL cells, respectively. Upregulated genes in the core region are highlighted in red, and upregulated genes in the periphery in gray. Avg.Exp., average expression; BMSC, bone marrow stromal cell; CLL, chronic lymphocytic leukemia; pct.exp, percent expression; PPI, protein–protein interaction.

To gain insight into the interaction between CLL B cells and BMSCs in the 3D system, the bulk RNA sequencing and the single‐cell RNA sequencing data were combined in a protein‐protein interaction network analysis. We extracted a list of putative interaction partners of CLL and BMSCs from the HIPPIE database (Human Integrated Protein‐Protein Interaction Reference)^46^ and examined which of these genes are differentially expressed in the BMSC clusters or between the two CLL cell cohorts (periphery and core). Within the BMSCs, we selected putative interaction partners that are differentially expressed among any of the clusters and are present in at least 10% of cells in any cluster. The expression levels of these genes in clusters 0 to 7 are depicted in Figure 5A. Additionally, putative interaction partners in CLL cells that are differentially expressed between the periphery and core region were selected from the HIPPIE list. Upregulated genes in the core are shown in the upper panel, whereas those upregulated in the periphery are shown in the lower panel (Figure 5B). The network in Figure 5C displays PPIs (solid lines) and self‐ligand interactions (squiggly lines). Putative interaction partners in BMSCs are represented by triangles, and those in CLL cells by circles, with red indicating upregulation in the core and gray indicating upregulation in the periphery. Remarkably, while differences in the expression of interaction partners are observed in cluster 5, no substantial differences in interaction partner expression are detected in the other clusters.

The putative PPI of IL12RB1 with IL23A, IL10RA with IL10, and MAP3K8 (mitogen‐activated protein kinase 8) with REL could indicate a connection among CLL B cells located in the core region. In the periphery, interactions of CLL cells might be mediated by AURKA, PLK1 (polo‐like kinase 1), BUB1B, BUB1, CDC20, and CCNB1 (cyclin B1). Since CDC20 is expressed not only in peripheral CLL cells but also in several BMSC clusters, this may point to a link between the two cell types. In addition, the putative interaction of BLNK (B cell linker) with BTK and PRKCB (protein kinase C beta) suggests a possible connection among CLL cells in the core region with ciBMSCs. Finally, the interaction between EGR1 and CEBPB (CCAAT/enhancer binding protein beta) represents a potential communication channel between CLL cells in the core and those in the periphery.

### Increased AP‐1 complex expression enhances survival of core‐located CLL cells and modulates ciBMSCs gene expression

**Figure 6:**
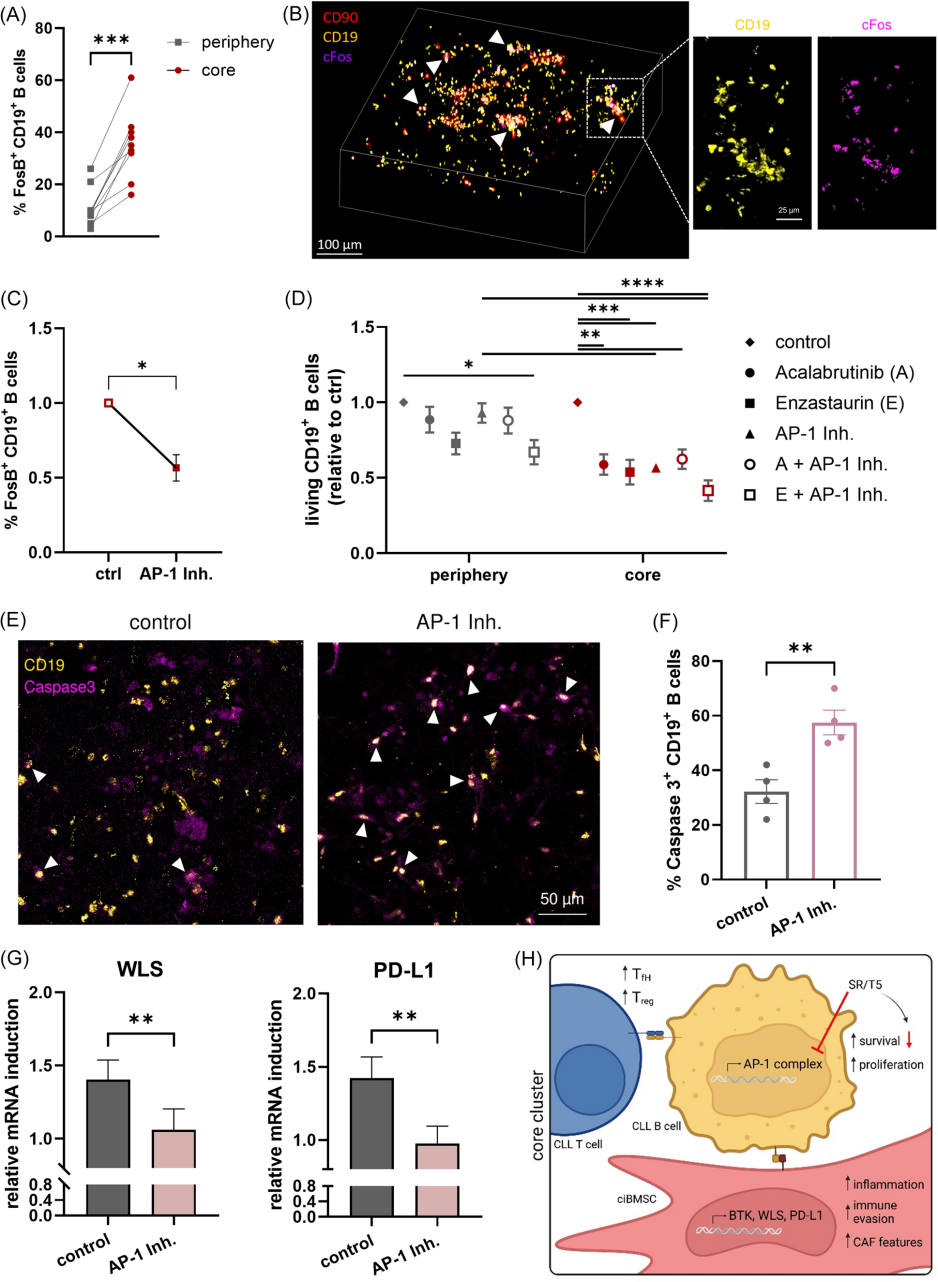
**Functional analysis of the AP‐1 complex in CLL B cells and effect of AP‐1 activity on BMSC gene expression**. **(A)** Flow cytometry analysis of FosB expression of CLL B cells in the periphery and the core region after 4 days of 3D co‐culture (*n* = 9). **(B)** *Z*‐stack reconstruction of immunofluorescence staining for BMSCs (CD90, red), B cells (CD19, yellow), and cFos (purple) showing an enhanced cFos expression within CLL cell clusters (white arrows) (number of stacks: 151; size of stacks: 1.06 µm). **(C)** Reduced number of FosB positive B cells after treatment with AP‐1 inhibitor SR11302 (10 µM) after 4 days in 3D co‐culture (*n* = 3). **(D)** Analysis of CLL cell viability in the periphery and the core region of 3D co‐culture after exposure to Acalabrutinib (10 µM), Enzastaurin (5 µM) or SR11302 (10 µM), and T5224 (10 µM), compared to the control (*n* = 4). **(E)** Immunofluorescence staining of the 3D co‐culture showing B cells (CD19) in yellow and apoptotic cells expressing Caspase 3 in purple. After treatment with a combination of SR11302 (10 µM) and T5224 (10 µM), a higher amount of apoptotic CLL B cells (white arrows) can be monitored compared to the control. **(F)** Quantification of the mean fraction of apoptotic cells in three images of the 3D model of four CLL patients. The number of Caspase 3^+^ CD19^+^ cells after AP‐1 inhibition was compared to the control. **(G)** Quantitative real‐time PCR analysis of WLS (left panel) and PD‐L1 (right panel) in BMSCs cultured in the 3D model for 4 days, treated with the AP‐1 inhibitor SR11302 (10 µM) compared to the control. **(H)** Schematic presentation of the CLL contact‐induced changes in the core region of the 3D co‐culture in T cells, B cells, and BMSCs. The figure was designed using BioRender. Bars indicate the standard error of the mean. *P < 0.05, **P < 0.01, ***P < 0.001, ****P < 0.0001 (paired *t*‐test, Holm–Sidak; D: two‐way ANOVA, Tukey). A, Acalabrutinib; AP, activator protein; CLL, chronic lymphocytic leukemia; E, Enzastaurin; Inh., Inhibitor; SR, SR11302; T5, T5224.

In this study, we demonstrate that several components of the AP‐1 complex are upregulated in the core region of the 3D model, suggesting that AP‐1 signaling is a potential target for CLL therapy. To assess the impact of the AP‐1 complex on disease progression, we investigated the effect of high FosB levels using functional assays. FACS analysis confirmed the differential expression of FosB between the peripheral and core regions (Figure 6A). 3D reconstruction of the co‐culture revealed a strong cFos signal (white arrows), particularly in areas where CLL B cells accumulate in clusters (Figure 6B). To demonstrate the importance of the AP‐1 complex for CLL B cell survival in the co‐culture system, CLL cells were exposed to various clinically used drugs alone or in combination with SR11302 and T5224, specific AP‐1 inhibitors.

The efficacy of AP‐1 inhibition was confirmed by a significantly lower number of FosB‐positive B cells after treatment (Figure 6C). Moreover, the efficient diffusion of the inhibitors into the internal niches of the 3D scaffold was demonstrated using fluorescently labeled dextrans resembling the size of SR and T5 (Supporting Information S1: Figure 5A). Following the addition of Acalabrutinib (BTK inhibitor), Enzastaurin (PKCβ inhibitor), or SR/T5 to the co‐culture, CLL cell survival was analyzed in the peripheral and core regions of the 3D culture system using flow cytometry (Figure 6D). In the periphery, only the combination of Enzastaurin with AP‐1 inhibitors results in a significantly reduced amount of viable CLL cells. In contrast, all inhibitors alone or in combination with SR/T5 led to CLL cell death in the core region. The strongest effect was induced by the combinatorial treatment with Enzastaurin and AP‐1 inhibitors. Remarkably, the comparison of AP‐1 complex inhibition in the two co‐culture regions revealed a significantly reduced survival rate of CLL cells exposed to SR/T5 alone or in combination with Enzastaurin in the core region.

The negative effect of AP‐1 inhibition on CLL cell survival was underlined by the visualization and subsequent quantification of Caspase 3^+^ apoptotic cells. Immunofluorescence staining revealed a significantly higher number of Caspase 3⁺ CLL B cells (white arrows) after AP‐1 inhibition (right panel) compared to the control (left panel) (Figure 6E). Quantitative image analysis confirmed this observation, showing a significantly increased number of apoptotic CLL B cells following treatment with AP‐1 inhibitors (Figure 6F). To investigate the influence of the AP‐1 inhibition on other cell types, 3D co‐cultures were treated with SR/T5, and the viability of periphery and core‐located CD3^+^ T cells, as well as BMSCs, was examined by FACS analysis. Whereas the T cells in the periphery are not affected, an increased amount of viable T cells can be observed in the core region after AP‐1 inhibition. In contrast, in BMSCs, the treatment with SR/T5 results in a reduced survival rate, contributing to CLL cell death (Supporting Information S1: Figure 5B). Furthermore, BMSC gene expression was assessed after 4 days of 3D co‐culture using qPCR (Figure 6G). Indeed, AP‐1 inhibition led to a significant reduction in WLS and PD‐L1 expression in BMSCs. Since these genes are upregulated in cluster 5, this finding suggests that core‐located CLL cells are closely associated with ciBMSCs. The crucial role of the AP‐1 complex in CLL cell survival and the regulation of BMSC gene expression underscores its potential as a therapeutic target.

## DISCUSSION

The impact of the TME on tumor development and progression has become increasingly recognized in recent years. In B‐cell malignancies, malignant cells actively manipulate their surroundings, resulting in TME‐mediated drug resistance leading to minimal residual disease (MRD). Cultivating primary cells in 3D conditions, which enable immune cells and BMSCs to interact in multiple dimensions, provides a more physiologically relevant model of dynamic cell‐cell communication occurring in vivo. These models also create natural gradients for oxygen and nutrient availability, further enhancing their physiological relevance.^47^, ^48^, ^49^ To further explore microenvironmental differences within the scaffold, we analyzed markers of hypoxia and apoptosis. While overall cell viability remained high, activated Caspase 3 staining revealed occasional apoptotic CLL cells primarily in the core (Supporting Information S1: Figurer 1C). Additionally, HIF1α expression indicated scattered hypoxic CLL cells, especially within cellular clusters in deeper scaffold regions (Supporting Information S1: Figure 1D). These findings support the presence of physiological gradients and stress‐related adaptations in the core niche. As previously shown, these 3D conditions can lead to altered therapeutic effects compared to 2D cultivation, particularly in core regions, where CLL B cells exhibit increased resistance to several inhibitors.^35^

In this study, we investigated the spatial distribution and interaction of CLL B cells, autologous T cells, and BMSCs within a scaffold‐based 3D model. Our findings provide important insights into the cellular organization and molecular mechanisms underlying drug resistance in CLL. We observed that BMSCs form a living scaffold, supporting the proliferation and survival of CLL B cells and T cells throughout the model (Figure 1). The critical role of BMSCs in sustaining CLL B cell survival through direct cell–cell contact is well established.^1^, ^2^, ^16^, ^50^, ^51^ Consistent with previous findings, a higher concentration of viable malignant B cells was detected in the core region.^35^ This observation highlights the role of BMSCs in establishing a protective niche, but also raises the question of how spatial positioning relative to BMSCs influences CLL cell fate. In addition to well‐known modulated genes in CLL B cells determined in 2D cell cultures, regulating, for example, the Notch pathway and the composition of the extracellular matrix,^1^, ^2^ our model identified differentially expressed genes regulating the AP‐1 complex in the core region, suggesting a previously unrecognized role of AP‐1 in CLL pathogenesis.^52^, ^53^ The AP‐1 complex, a transcription factor network involved in proliferation, survival, and oncogenesis, is a heterodimer composed of members of the Fos family (cFos, FosB, Fra‐1, and Fra‐2) and the Jun family (cJun, JunB, and JunD). It is highly oncogenic, and its deregulation alone is sufficient for neoplastic transformation.^54^ Constitutive AP‐1 expression is a characteristic of Hodgkin's lymphoma (HL) and anaplastic large cell lymphoma (ALCL), where it drives a common oncogenic transcriptional program.^53^, ^55^ Notably, AP‐1 components are silent or weakly expressed in circulating CLL cells but are highly induced in lymph node‐resident CLL cells,^56^ supporting our findings that CLL cells become activated within the 3D model (Figures 1, 2A,B, and 6). Observations identifying a potential pathogenic role for transcription factor dysregulation in CLL, including AP‐1 imbalances, the epigenetic program of B cells.^57^ Therefore, our data suggest that AP‐1 contributes to the establishment of a protective niche within the TME, further reinforcing its potential as a therapeutic target.

Beyond CLL cells, T cells were distributed in both the peripheral and core regions, where they directly interacted with malignant B cells. Of interest, T follicular helper (T_fH_) cells were also supported in our 3D setting, especially in the core region (Figure 2I). The aspect that the accumulation of T_fH_ cells is concomitant with the expansion of CLL cells with a functional ability to promote CLL proliferation, while also CLL cells can activate the T_fH_ cells, vice versa, suggests that the 3D model may provide a conducive environment for T_fH_ cells resembling the in vivo situation.^58^ Moreover, activated regulatory T cells (T_reg_) are crucial for CLL progression, as shown in TCL1 leukemia models, where T_regs_ create an immunosuppressive environment that supports leukemia survival.^59^ As described in other studies, T_reg_ levels were elevated in patients with CLL compared to matched controls, and a progressive increase of T_reg_ was detected in advanced stages of the disease.^60^, ^61^, ^62^ The results assessed the regulatory function of CLL B cells with a suppressive activity on Th1 cellular response and the induction of CD4^+^ FOXP3^+^ T_regs_.^63^ Importantly, our model recapitulates this pattern, with CD4⁺ FOXP3⁺ Tregs enriched in the core region, where viable and transcriptionally altered CLL B cells are concentrated. This alignment with in vivo observations further supports the model's physiological relevance. Proliferating CLL B cells interact with autologous T cells. Activated T cells proliferate and show functionality via Granzyme B expression and cytokine secretion (Figure 2G,H). However, they also show signs of replicative senescence via CD57 (Figure 2I) and a trend toward T cell exhaustion (PD1^+^/TIM3^+^/LAG3^+^, Figure 2F), especially in the core region. Notably, T‐cell proliferation is enhanced in our model and is driven by the combination of the 3D structure and direct contact with CLL cells, but not by the scaffold or BMSCs alone (data not shown). Interestingly, Kinney et al. demonstrated that a subpopulation of CD57^+^ CD27^−^/CD28^−^ T cells from head and neck cancer patients retained the ability to proliferate, indicating a highly differentiated effector memory T cell phenotype with sustained proliferative capacity.^40^ This suggests that despite senescence‐associated markers, T cells within our 3D system may still retain functional activity and proliferative potential in response to CLL interactions. Overall, the expression of homing and T cell interaction markers on CLL B cells, as well as the activation/exhaustion profile of the autologous T cells, predicts that our 3D setting is a relevant in vitro model to mimic the cellular communication between different cell types of the TME.

Single‐cell RNA sequencing provided detailed insights into BMSC heterogeneity. Notably, we identified a novel stromal cluster (ciBMSCs, cluster 5), which emerged exclusively following contact with CLL B cells (Figure 6H). This cluster exhibited hallmarks of chondrocyte‐ and CAF‐like phenotypes.^1^, ^2^, ^10^, ^38^ Genes differentially expressed in ciBMSCs were involved in lipid metabolism^64^ and cholesterol biosynthesis, pathways also relevant in multiple myeloma.^65^ Importantly, ciBMSCs exhibit immunosuppressive properties, further indicating that CLL cells do not randomly reprogram BMSCs but instead induce specific transcriptional changes that favor tumor survival. This aligns with our observation that AP‐1 complex components are upregulated in core‐resident CLL cells (Figure 3). Focusing the gene regulation in these distinct ciBMSCs during contact with CLL cells, immune evasion mechanisms triggered by cancer cells seem to be not only an important point of research in the TME of solid tumors.^66^, ^67^, ^68^, ^69^ Moreover, using the HIPPIE database to score and annotate possible protein‐protein interactions, we predicted several interaction partners between CLL B cells and BMSCs. Notably, BTK signaling was identified as a key pathway, which is well known for its role in B cell maturation, but it is also critical for cancer cell proliferation and evasion of cancer‐killing cells.^43^ Most BTK studies have focused on B cells. BTK inhibitors, such as Acalabrutinib and Ibrutinib, are FDA‐approved for CLL and mantle cell lymphoma, with Acalabrutinib showing improved specificity. However, some patients relapse due to MRD.^70^, ^71^ This suggests that BTK inhibition alone is insufficient to eradicate all malignant cells within protective niches in the TME. Of note, the use of BTK inhibitors to target the TME as a way to combat solid tumors is under investigation.^72^

Additionally, CENPA overexpression was observed in ciBMSCs. It has been identified as a feature of many solid cancers, including breast, bone, and liver cancer.^73^, ^74^, ^75^ Recent studies uncovered that CENPA overexpression can reprogram cell fate and impact 3D nuclear organization in cancer.^76^, ^77^, ^78^ To our knowledge, this is the first time that CENPA regulation is reported in the context of TME in hematological malignancies, suggesting a previously unrecognized role of CENPA in the CLL TME.

To rule out technical limitations in compound delivery, we performed dextran diffusion assays confirming that both stromal and leukemic compartments are accessible across the full scaffold depth (Supporting Information S1: Figure 5A), supporting the functional interpretation of region‐specific drug resistance.

While our model offers significant insights, it has limitations. The complexity of the TME, including the full immune repertoire and the diversity of stromal phenotypes in vivo, cannot be fully captured in vitro. In particular, the functional relevance of the T cell exhaustion phenotype observed in the core region remains to be elucidated. To address this, future experiments will include TCR spectratyping to assess clonality and antigen specificity of T cells within the scaffold. Another key question relates to the plasticity of ciBMSCs. We are currently planning targeted experiments to determine whether their reprogrammed state is reversible, for example, through the removal of CLL cells or the inhibition of AP‐1 signaling. Preliminary experiments suggest that this reprogramming involves both reversible and stable components. Following immune cell removal, FACS analysis showed a reduction in BTK and WLS expression, whereas PD‐L1 and POU3F1 levels remained stable (Supporting Information S1: Figure 4). These findings imply that ciBMSCs retain certain features independent of ongoing leukemic contact, while others may reflect a dynamic adaptation to the surrounding microenvironment.

While we did not include direct comparisons to 2D cultures in this study, we have previously shown that spatial positioning within the scaffold leads to distinct drug responses not observed under 2D conditions.^35^ Building on these findings, our present work focuses specifically on the spatial heterogeneity and microenvironmental crosstalk that emerge within the 3D setting. Despite these limitations, our 3D co‐culture model is the first to use primary autologous immune cells in a spatially defined system, allowing the characterization of distinct tumor microenvironmental niches. It offers a robust platform to explore spatially organized immune–stromal–tumor interactions and to guide therapeutic strategies in CLL.

In conclusion, our 3D model serves as a powerful tool for studying the interactions between CLL B cells, BMSCs, and T cells within a physiologically relevant context. The insights gained from this study could pave the way for future research and potential therapeutic advancements in CLL.

## AUTHOR CONTRIBUTIONS

**Jana Lindacher**: Writing—original draft; methodology; formal analysis; investigation; visualization. **Anne Hartebrodt**: Writing—original draft; methodology; software; formal analysis. **Janin Dingfelder**: Methodology; Writing—review and editing. **Pascal Lukas**: Methodology; software; Writing—original draft. **Laeschkir Würthner**: Software; Writing—original draft; methodology. **Simon Völkl**: Resources. **David B. Blumenthal**: Writing—review and editing; conceptualization. **Frederik Graw**: Writing—review and editing; conceptualization. **Kerstin Amann**: Resources. **Manuela Krumbholz**: Writing—review and editing. **Martina Haibach**: Resources. **Jochen Wilke**: Resources. **Andreas Mackensen**: Writing—review and editing; Conceptualization. **Gloria Lutzny‐Geier**: Conceptualization; funding acquisition; Writing—original draft; project administration; supervision; validation; data curation.

## CONFLICT OF INTEREST STATEMENT

The authors declare no conflict of interest.

## ETHICS STATEMENT

All procedures were conducted in accordance with the Good Clinical Practice guidelines of the International Council for Harmonization and covered by license number: 219_14B, addendum 59_17 Bc. All participants gave written informed consent according to CARE guidelines and in compliance with the Declaration of Helsinki.

## FUNDING

The study was supported by the Deutsche Forschungsgemeinschaft (DFG)–Project number LU2181/1‐2 and the Sanderstiftung (2021.059.1, 2020.045.1) to G. L.‐G, and the Hightech Agenda Bavaria and BMBF (031L0293E) to F.G. Cell collection, RNA sequencing, and immunofluorescence analyses have been performed at the Core Units “Cell Sorting and Immunomonitoring”, “Next Generation Sequencing”, and the “Optical Imaging Center” Erlangen.

## Supporting information

Supplementary Material Revision1.

## Data Availability

The data that support the findings of this study are available from the corresponding author upon reasonable request.
