# Supplementary material for: 3D Tumor microenvironment interaction reveals AP‐1 complex regulation and contact‐mediated reprogramming of bone marrow stromal cells in chronic lymphocytic leukemia
Source: Hemasphere. 2025 Aug 13;9(8):e70199. doi: 10.1002/hem3.70199 (PMC12348881; doi:10.1002/hem3.70199)
Supplement: Supplementary file 1 — Supplementary Material Revision1. [file HEM3-9-e70199-s001.docx]

**Supplement 1: Supplemental data figure 1**


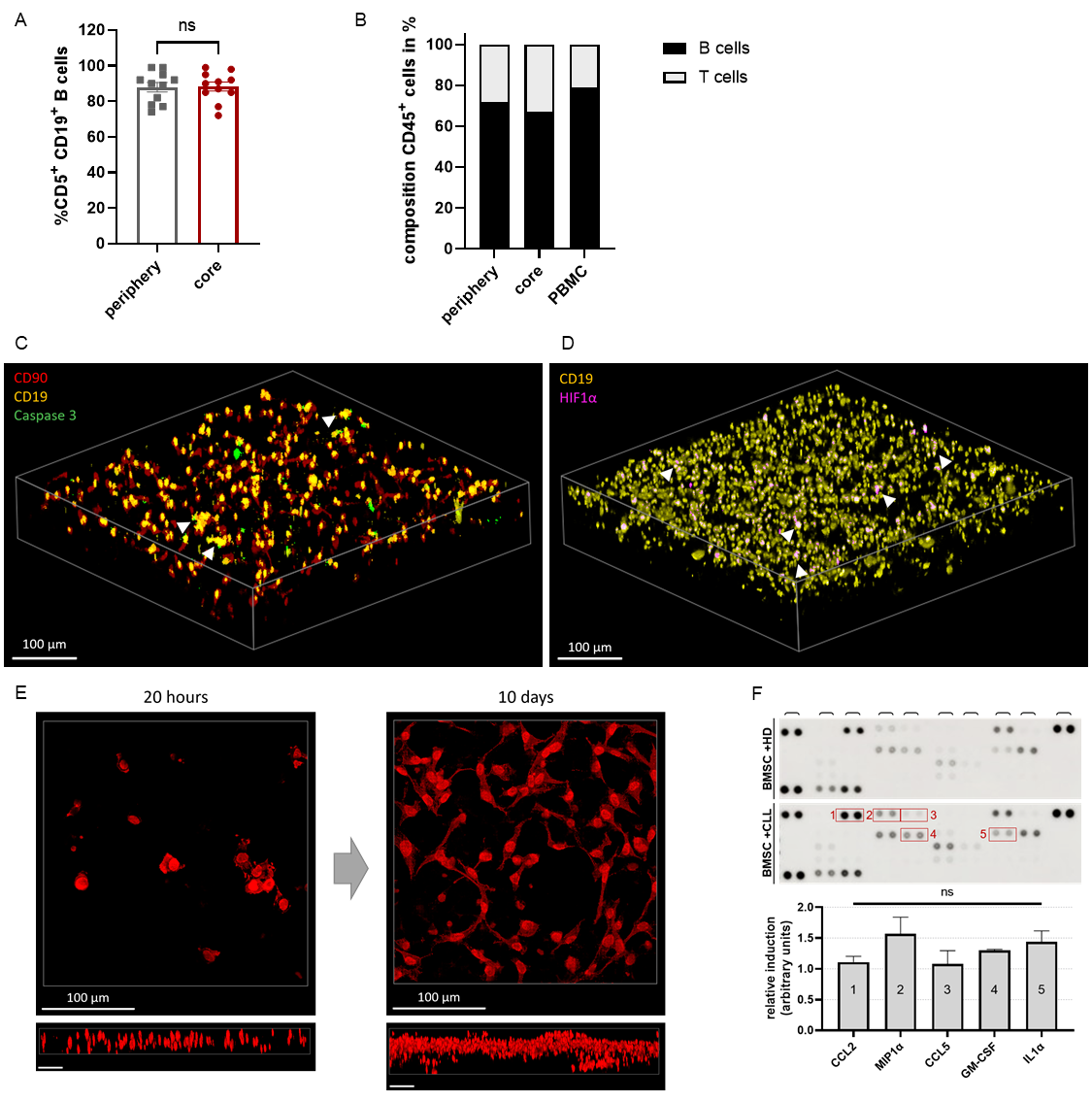


**Supplementary Figure S1: Characterisation of the scaffold-based 3D cell culture system.** (A) The number of CD5^+^ CD19^+^ B cells was measured after 4 days of co-culture in the peripheral and core region of the 3D scaffold by FACS analysis (n=11). (B) Comparison of initial composition of T and B cells in PBMCs and ratio of T and B cells in the periphery and the core region of the 3D cell culture system reveal equal cell amounts in all 3 conditions (n=12). (C, D) 3D reconstruction of an immunofluorescence staining of the scaffold after 4 days of co-culture with CD90^+^ BMSCs marked in red and CD19^+^ CLL cells in yellow. Apoptotic cells are defined by the expression of Caspase 3 (green) and hypoxic cells by the expression of HIF1α (purple) (number of stacks: C 104, D 116; size of stacks: 1.06 µm). (E) Immunofluorescence images of BMSCs cultured in the 3D scaffold for 20 hours versus 10 days stained with DAPI and phalloidin visualize the expansion of BMSCs and the transition to a stellate morphology. (F) Cell-free supernatants of 3D co‑cultures after 4 days with B and T cells from CLL patients or healthy donors (HD) were analysed using a human cytokine array (n=3). A representative blot of each culture condition is presented (upper panel). The relative induction (CLL/HD) of detected cytokine levels released in the 3D culture during CLL contact compared to HD is shown (lower panel). ns: not significant (unpaired t-test).

**Supplement 2: Supplemental data figure 2**

**
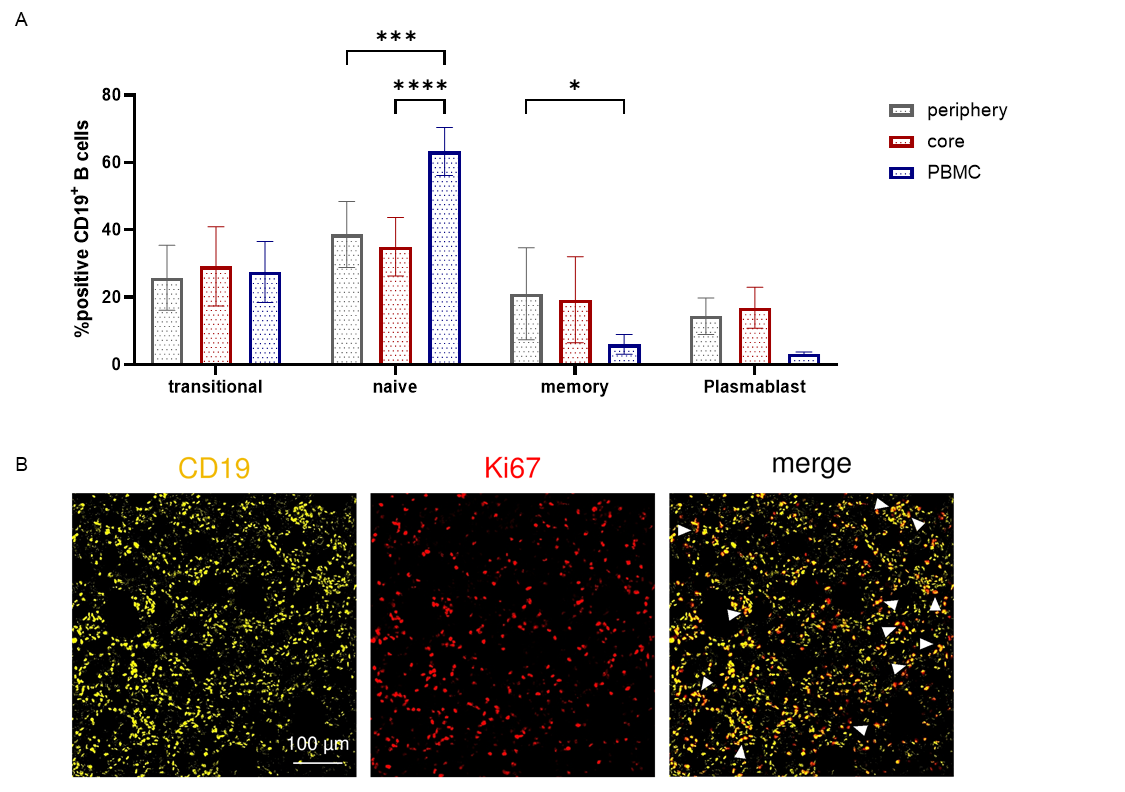
**

**Supplementary Figure S2: Differentiation and proliferation of CLL B cells in the 3D co-culture** (A) Differentiation stages of CD19^+^ B cells in PBMCs or in the periphery and the core region of the 3D co-culture. A significant higher level of naïve B cells and a reduced amount of memory B cells can be observed in PBMCs (n=4-8). *p < 0.05, ***p < 0.001, ****p < 0.0001 (2way ANOVA). (B) Co-localisation (white arrows, merge) of CD19 (yellow) and Ki67 (red) expression in immunofluorescence staining visualize proliferation of malignant B cells in the 3D model.

**Supplement 3: Quality metrics cell cluster**


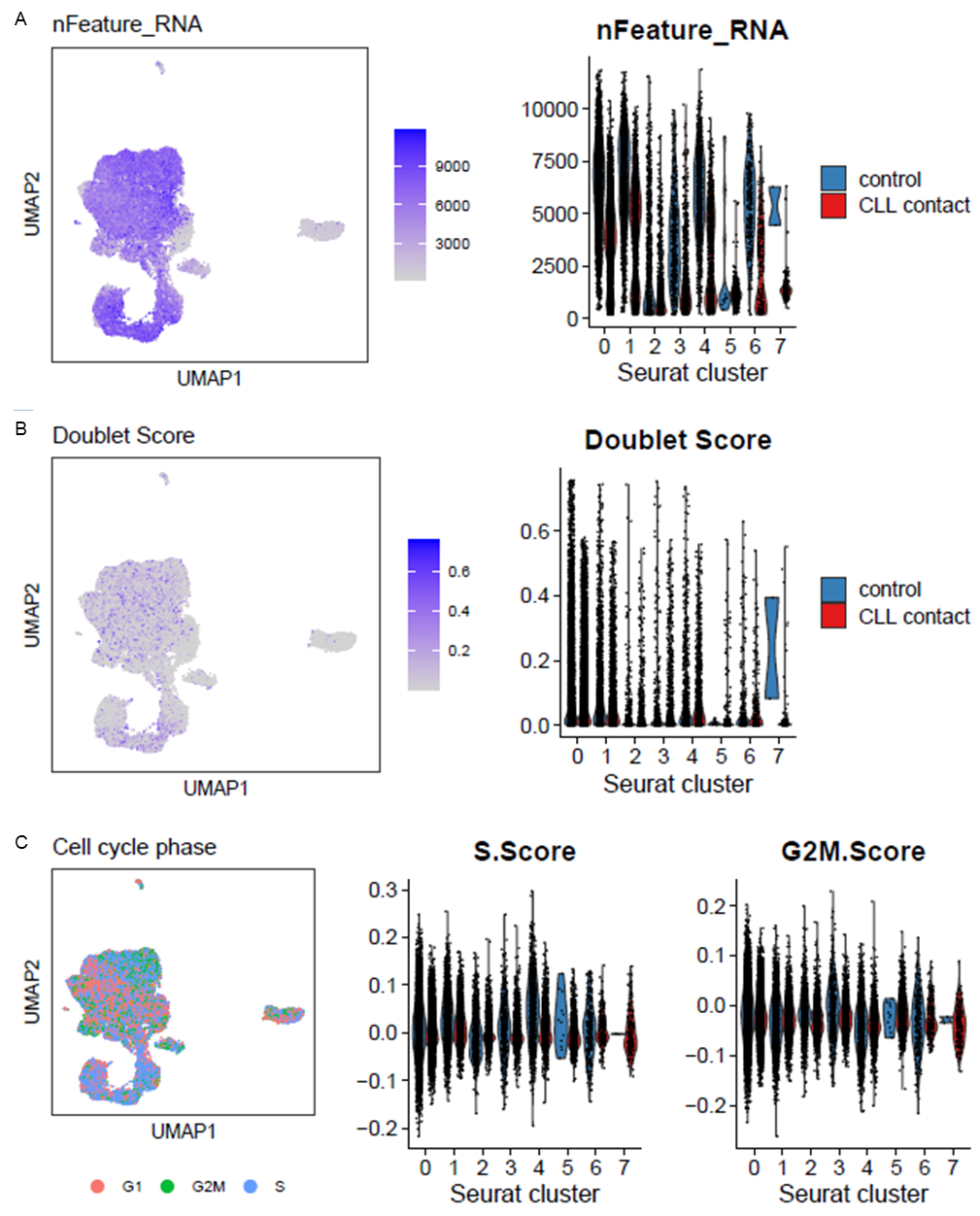


**Supplementary Figure S3: Quality metrics for all 8 cell clusters.** (A) Sequencing depth is comparable in all clusters. (B) Doublets are only detected in minor quantities. (C) Cell cycle is overall not a source of confounding.

**Supplement 4: Supplemental data figure 4**

**
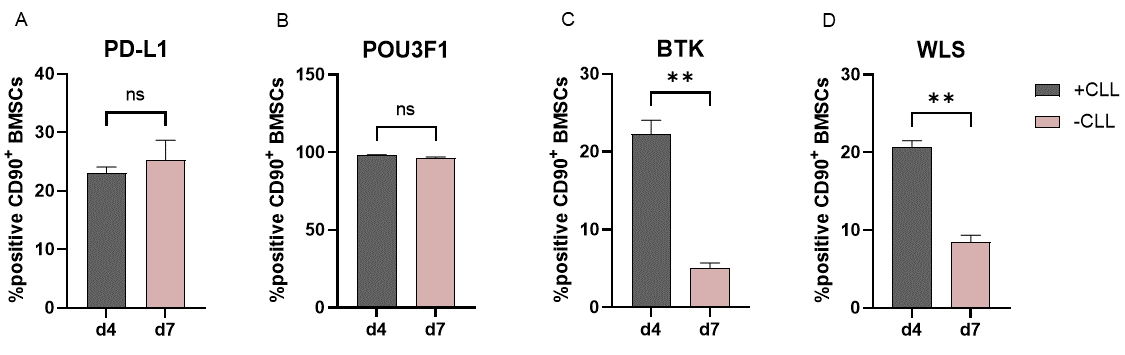
**

**Supplementary Figure S4: Plasticity of reprogrammed ciBMSC state.** The proportion of BMSCs expressing (A) PD-L1, (B) POU3F1, (C) BTK and (D) WLS was examined by FACS analysis and compared after 4 days of 3D co-culture and after removal of the immune cells following cultivation for further 3 days (n=4). **p < 0.01(paired t-test, Holm-Sidak).

**Supplement 5: Supplemental data figure 6**

**
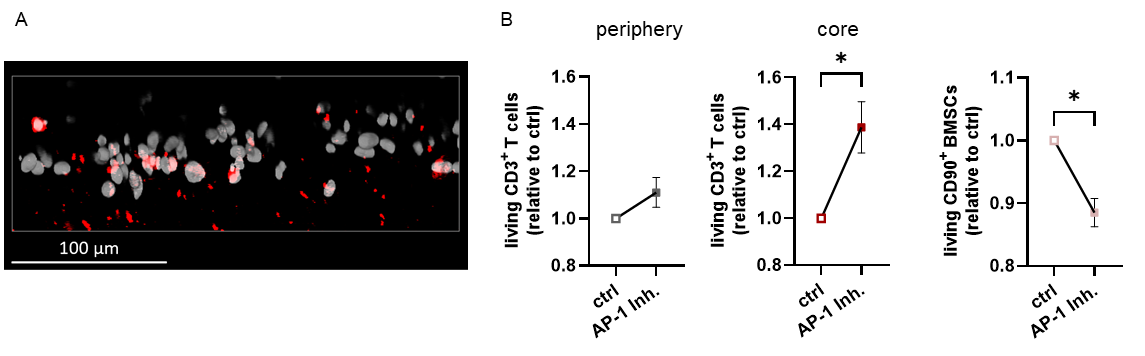
**

**Supplementary Figure S5: Diffusion of inhibitors and impact of AP-1 inhibition on T cells and BMSCs.** (A) Z-stack reconstruction of immunofluorescence images of 3D scaffold cultured with BMSCs (gray) for 10 days reveal an efficient diffusion of fluorescently labelled dextran (red) resembling the size of SR and T5 into internal areas of the scaffold. (B) Impact of AP-1 inhibition on viability of CD3^+^ T cells in the periphery and the core region as well as CD90^+^ BMSCs after 4 days of 3D co-culture was assessed by flow cytometry (n=4). *p < 0.05 (paired t-test).

**Supplement 6: patient list**

**Supplement 7: antibody list**

**Supplement 8: Quality control Single-cell RNA sequencing**


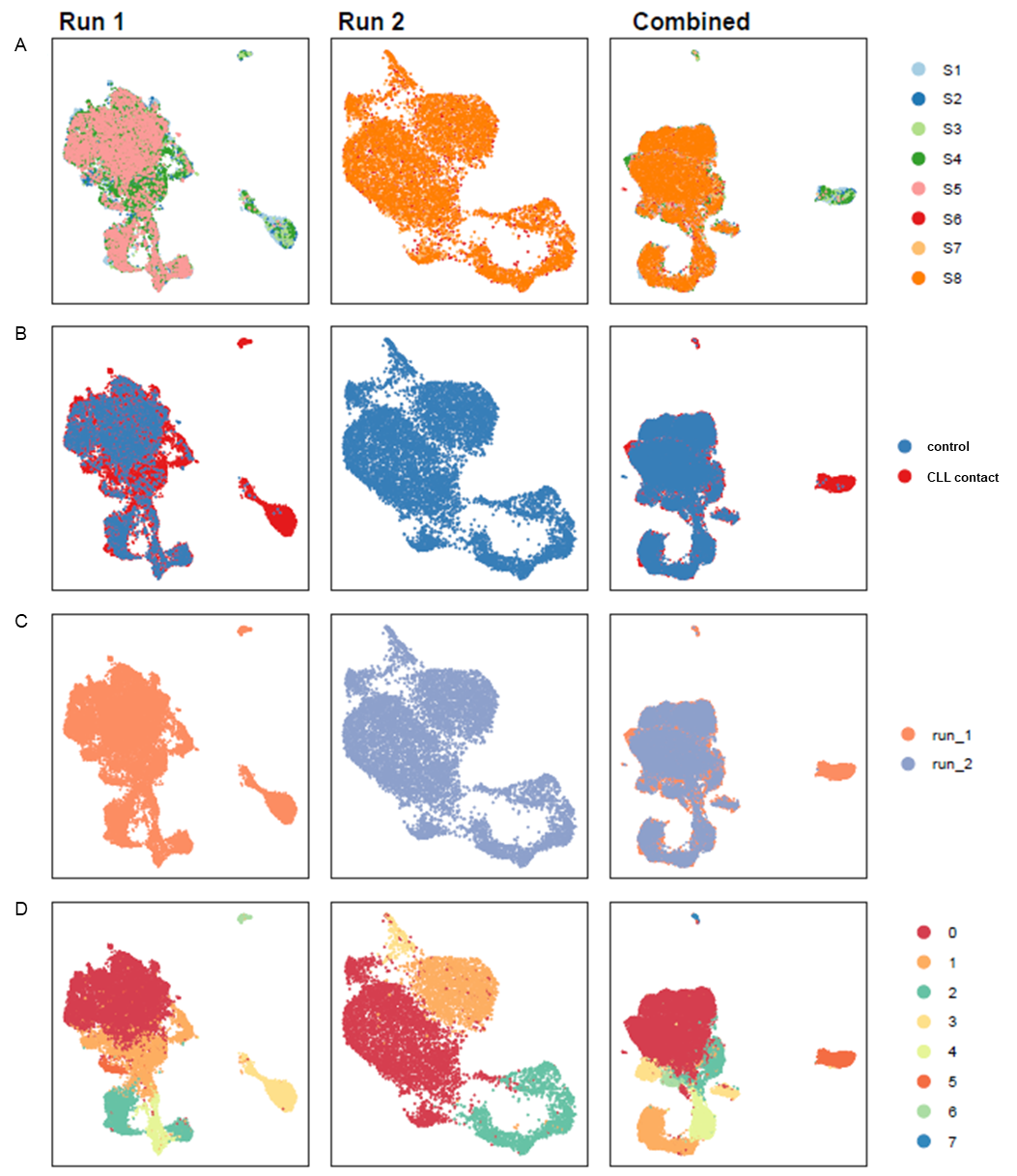


**Supplementary Figure S8: Quality control plots for single cell expression data.** UMAPs and clustering have been computed using different subsets of data titled Run 1 (S1‑S5), Run 2 (S6‑S8) and Combined. A comparison of the data subsets of (A) all 8 samples S1-S8, (B) cells with or without contact to CLL cells (C) the two different runs and (D) the 8 clusters are shown.

**Supplement 9: Abbreviations**

ALCL Anaplastic Large Cell Lymphoma

ALL Acute Lymphoblastic Leukemia

AML Acute Myeloid Leukemia

AP-1 Activator‑Protein 1

BMSC Bone Marrow Stromal cells

ciBMSC contact-induced Bone Marrow Stromal cells

CAF Cancer-associated Fibroblasts

CLL Chronic Lymphocytic Leukaemia

DLBCL Diffuse Large B-cell Lymphoma

FACS Fluorescence Activated Cell Sorting

HD Healthy donor

HL Hodgkin`s Lymphoma

Inh Inhibitor

MM Multiple Myeloma

MRD Minimal‑residual disease

NES Normalised Enrichment Score

NKT Natural Killer T Cell

PBMC Peripheral Blood Mononuclear Cell

PBS Phosphate‑buffered saline

PPI Protein‑Protein interaction

SEM Standard error of the mean

TBS-T TRIS‑buffered saline with Tween20

TME Tumor Microenvironment

UMAP Usine Moderne d'Applications Plastiques
